# Anti-Inflammatory Activity of Compounds Isolated from Plants

**DOI:** 10.1100/tsw.2001.77

**Published:** 2001-11-29

**Authors:** R.M. Perez G.

**Affiliations:** Laboratorio de Investigacion de Productos Naturales, Escuela Superior de Ingenieria Quimica e Industrias Extractivas, IPN, Punto Fijo 16 Col. Torres Lindavista cp 07708, Mexico D.F., Mexico

**Keywords:** anti-inflammatory activity

## Abstract

This review shows over 300 compounds isolated and identified from plants that previously demonstrated anti-inflammatory activity. They have been classified in appropriate chemical groups and data are reported on their pharmacological effects, mechanisms of action, and other properties.

## INTRODUCTION

Major research efforts to find new therapeutic agents for a variety of inflammatory skin diseases are motivated mainly by the medical need to find drugs with fewer side effects than the ones currently employed. During the more than 35 years since the introduction of cortisone and phenylbutazone for the treatment of rheumatic diseases, a number of new compounds have been studied for anti-inflammatory activity. Some are also useful in the treatment of fever and gout.

In the treatment of rheumatic diseases, basically two types of drugs are used, nonsteroidal anti-inflammatory drugs (NSAIDs) and disease-modifying antirheumatic drugs (DMARDs). NSAIDs mainly inhibit the cyclo-oxygenase pathway of the arachidonic acid cascade, preventing the formation of proinflammatory prostaglandins. DMARDs are immunomodulators claimed to regulate a distorted immune system[1].

A large number of compounds of varied chemical structures isolated from medicinal plants have been shown to possess anti-inflammatory activity. In this review, over 300 compounds isolated from plants with anti-inflammatory activity are shown. This article is based on bibliographic research of Chemical Abstracts from 1950-2000. It can be helpful to researchers in the study of anti-inflammatory substances from plants. This also may serve as a guide for pharmacologists studying the mechanisms of action and anti-inflammatory effects of these substances. For only a few anti-inflammatory compounds isolated from plants has the principal mechanism of action been determined definitively.

## ALKALOIDS AND NITROGEN COMPOUNDS

**1**

**3-Acetylaconitine**

An isoprenoid pathway-derived alkaloid isolated from *Aconitum flavum* Hand-Mazz and *A. pendulum* Busch, (Ranunculaceae). Increased vascular permeability induced by acetic acid or histamine is considerably inhibited by 3-acetylaconitine. Edema produced by carrageenan or fresh egg white is also inhibited[2].

**2**

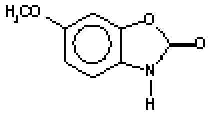

**Betonicine**

A piperidine alkaloid found to significantly inhibit carrageenan-induced hindpaw edema[3]. Isolated from *Betonica officinalis*, *Marrubium vulgare*, *Stachys sylvatica* (Labiatae), *Achillea moschata,* and *A. millefolium* (Compositae).

**3**

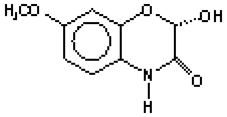

**Benzoxazinoid**

The roots of *Coix lachryma-jobi* have been used in China for the treatment of rheumatism and neuralgia. Two benzoxazinoid compounds isolated from the roots of *C. lachryma-jobi* L. (Graminae) showed 85.5 and 47.3% inhibitory activity of histamine release, respectively[4].

**4**

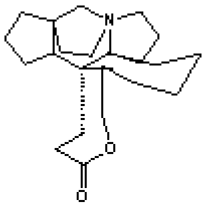

**Bukittinggine**

The major alkaloid of *Sapium baccatum* Roxb. (Euphorbiaceae). Has anti-inflammatory activity on carrageenan-induced hindpaw edema and adjuvant-induced arthritis in rats[5]. This plant is used by indigenous people of West Sumatra, Indonesia in folk medicine for remedies to treat bronchial asthma and to relieve pain.

**5**

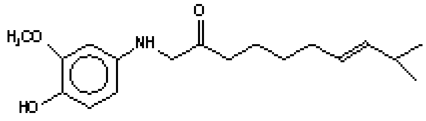

**Capsaicin**

A pungent principle of various *Capsicum* spp. (Solanaceae). Used as a selective probe for studying neurogenic inflammation and the role of nociceptors in human physiology[6].

**6**

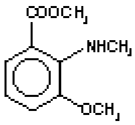

**Damascenine**

Found in seeds of *Nigella damascena* and *N. arvensis* (Ranunculaceae). Inhibits induced edema formation in the paw[4].

**7**

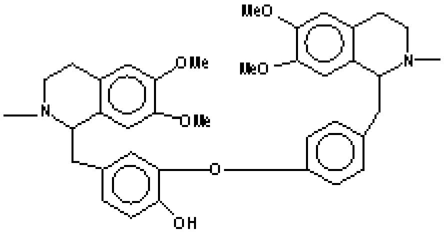

**Dauricine**

In traditional Chinese medicine, dauricine, an alkaloid isolated from *Menispermun dauricum* DC. (Menispermaceae) roots, has been used for testing rheumatism and is toxic ($LD_{50}$ i.p. in mice 6 mg/kg$^{-1}$)[7].

**8**

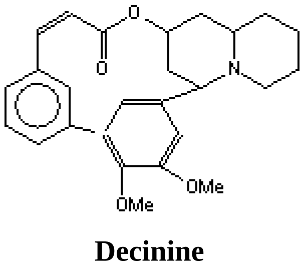

**Decinine**

Found in *Lythrum lanceolatum* L. (Lythraceae). Has anti-inflammatory activity[8].

**9**

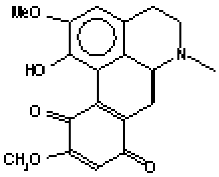

**Demethylsonodione**

Found in the stem bark of *Hernandia sonora* L. (Hernandiaceae). Showed moderate antiplatelet aggregation activity *in vitro*[9].

**10**

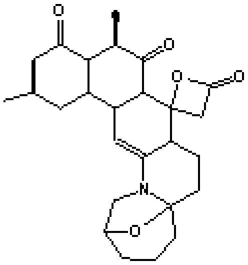

**Deoxyzoanthenamine**

Isolated from an unidentified colonial Zoanthid of the genus *Zoanthus*. A potent anti-inflammatory and analgesic agent[8].

**11**

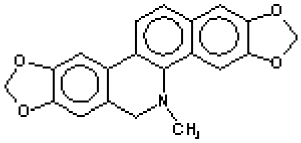

**Dihydrosanguinarine**

A benzophenanthridine alkaloid found in *Fumaria parviflora* (Papaveraceae). Activity and uses similar to those of sanguinarine. Used as antiseptic and anti-inflammatory agents[3].

**12**

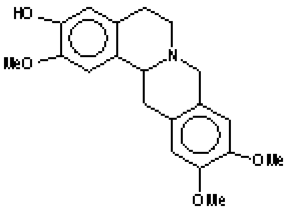

**Discretine**

Found in *Xalopia discreta.* Claimed to have analgesic, anti-inflammatory activity[10].

**13**

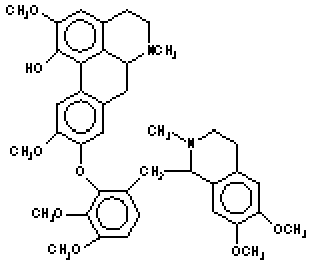

**Fetidine**

An isoquinoline alkaloid that is anti-inflammatory, hypotensive, and depresses nervous activity in mice[11]. Isolated from *Thalictrum foetidume* (Ranunculaceae) leaves.

**14**

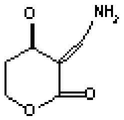

**Gentianaine**

Showed low anti-inflammatory activity in carrageenan-induced hindpaw edema[12]. Isolated from *Gentiana caucasa* (Gentianaceae) leaves.

**15**

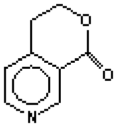

**Gentianadine**

A sesquiterpene alkaloid found in the aerial parts of *Gentiana turkestanorum*, *G. olgae*, and *G. olivieri* (Gentianaceae). Shows anti-inflammatory and muscular relaxant actions[12].

**16**

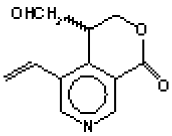

**Gentianamine**

Showed anti-inflammatory activity in carrageenan-induced hindpaw edema[12]. Isolated from leaves of *Gentiana olivieri* and *G. turkestanorum* (Gentianaceae).

**17**

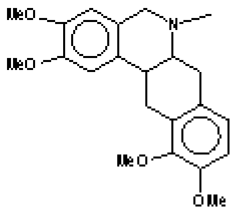

**Glaucine**

Found in a wide variety of genera in the Annonaceae (*Alphonsea*, *Annona*, *Polyalthia*, and *Uvaria*). Shows analgesic and anti-inflammatory activity[8].

**18**

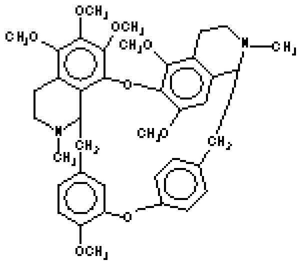

**Hernandezine**

Isolated from *Stephania hermandifolia* (Menispermaceae) and *Thalictrum simplex* (Ranunculaceae) leaves and studied in the rat paw carrageenan and the cotton pellet granuloma[13].

**19**

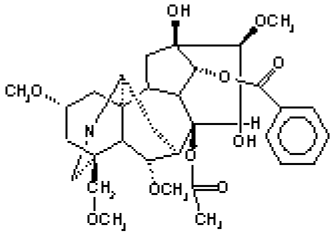

**Hypaconitine**

Betalain alkaloid obtained from *Aconitum collianthum*, *A. carnichaeli*, and *A. napellus* (Ranunculaceae). Showed significant inhibitory activity against carragenan-induced edema[14].

**20**

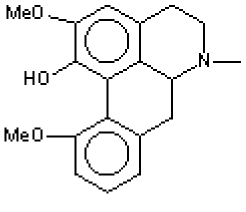

**Isothebaine**

Isolated from *Papaver orientale* and *P. bracteatum* (Papaveraceae). Shows analgesic and anti-inflammatory activity[8].

**21**

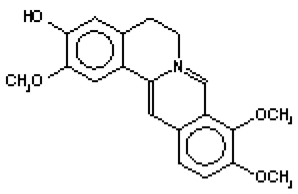

**Jatrorrhizine**

Alkaloid isolated from methanol extract of cultured *Plagiorhegma dubium* Maxim (Berberidaceae). Has shown anti-inflammatory activity[15].

**22**

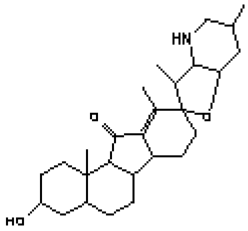

**Jervine**

Some *Veratrum* alkaloids, namely jervine (and jervine derivatives) and protoveratrine, exhibited anti-inflammatory activity when administered subcutaneously. They decreased both exudative and proliferative phases consequent to subcutaneous implantation of wool pellets[16]. Isolated from *Veratrum lobelianum* (Liliaceae) leaves.

**23**

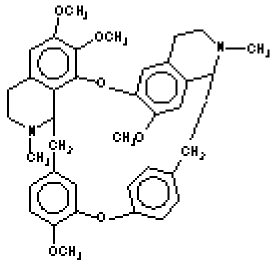

**(+)-O-Methylthalicberine (or Thalmide)**

Another alkaloid isolated from leaves of *Lictrum* spp. (Ranunculaceae) and *Berberis laurina* (Berberidaceae). Prevented the development of the paw carrageenan edema[17].

**24**

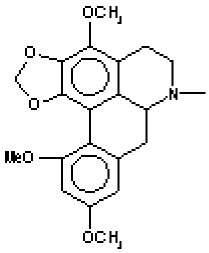

**Ocoteine**

Occurs in *Ocotea puberula* (Lauraceae). Shows analgesic, antimicrobial, and anti-inflammatory activity[8].

**25**

$CH_3$-$(CH_2)_{14}CONHCH_2CH_2OH$

**Palmidrol**

Occurs in soya bean lecithin. Shows analgesic, antimicrobial, and anti-inflammatory activity[8].

**26**

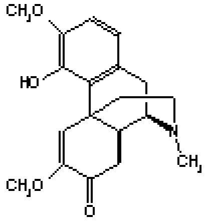

**Sinoacutine**

*Antizoma angustifolia* Burch. (Menispermaceae) leaves yielded a morphinandienone-type alkaloid sinoacutine in 2.8% yield. Alkaloid exhibited slight anti-inflammatory activity[18].

**27**

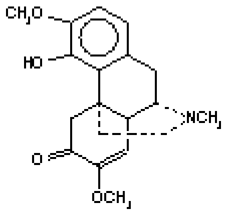

**Sinomenine**

A major alkaloid of the traditional Chinese drug plant *Sinomenium acutum* Thunb. Has marked anti-inflammatory activity[19].

**28**

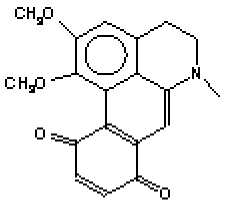

**Sonodione**

Found in the stem bark of *Hernandia sonora* L. (Hernandiaceae). Showed moderate antiplatelet aggregation activity *in vitro*[9].

**29**

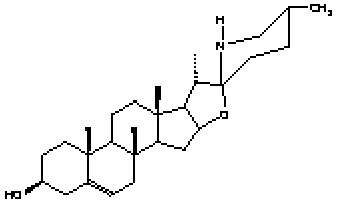

**Solasodine**

Caused significant dose-dependant inhibition of carrageenan-induced paw edema[20]. Isolated from *Solanum lacintatum* (Solanaceae) leaves.

**30**

$C_{14}H_{23}NO$

**Spilanthol**

Alkaloid isolated from the roots of *Sapilanthes olereaceae*. Has anti-inflammatory activity on carrageenan-induced hindpaw edema and adjuvant-induced arthritis in rats[6].

**31**

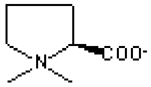

**Stachydrine**

Found in *Stachys* spp. (Leguminosae). Widely used against rheumatism and many other diseases. Also a systolic depressant[8].

**32**

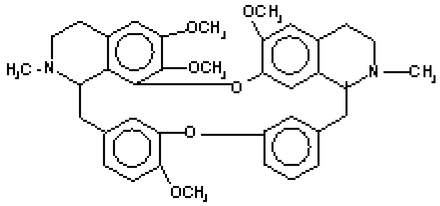

**Tetrandrine**

Isolated from root of *Stephania tetrandra* S. Moore. Exhibits definitive antiphlogistic action on formaldehyde-induced arthritis of rats[21].

**33**

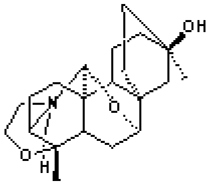

**Thalicsiline**

A spiradine-type C20-diterpenoid alkaloid isolated from the roots of *Thalictrum sessile* Hayata (Ranunculaceae). Has anti-inflammatory activity[22].

**34**

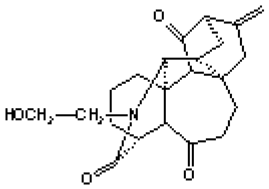

**Thalicsessine**

The first ajaconine-type C20 diterpene alkaloid having an oxygen function at C-20. Isolated from *Thalictrum sessile* (Ranunculaceae). Showed 40% reduction of carrageenan-induced inflammation at 20 mg/kg[12].

**35**

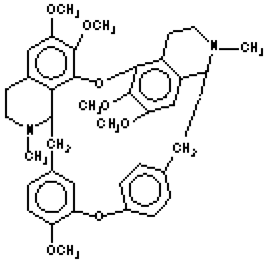

**Thalidasine**

The leaves of *Thalictrum dasycarpum* (Ranunculaceae) are reported to be applied to rheumatic swelling of joints and sprains. Exhibited anti-inflammatory activity in carrageenan-induced hindpaw edema[13].

**36**

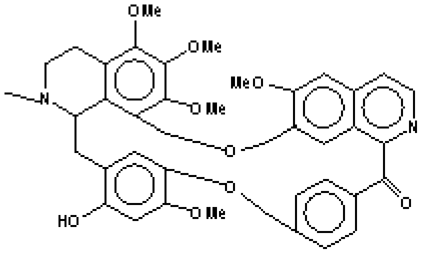

**Thalictrinine**

Occurs in *Thalictrum rochebrunianum* (Ranunculaceae). Shows strong anti-inflammatory activity and weak antitumor agent[8].

**37**

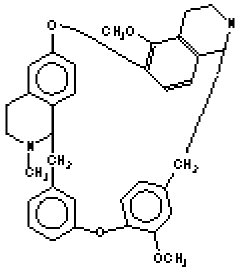

**Thalmine**

Showed anti-inflammatory activity in several experimental models of inflammation in animals. Antitumor activity shown against ascites lymphoma in rats and mice[13]. Isolated from *Thalictrum* spp. (Ranunculaceae) leaves.

**38**

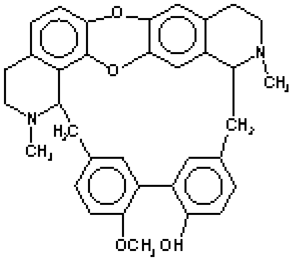

**Trilobine**

**39**

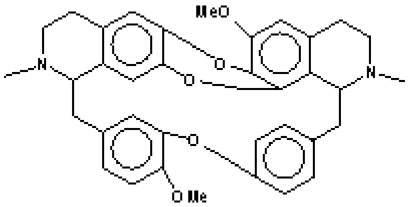

**Isotrilobine**

Two alkaloids, trilobine and isotrilobine, isolated from roots of *Cocculos trilobus* (menispermaceae) showed inhibitory effect against cotton pellet and adjuvant arthritis in rats[23].

**40**

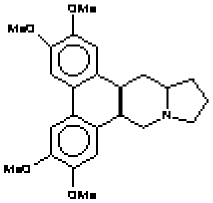

**Tylophorine**

Inhibited the primary and secondary responses of adjuvant-induced arthritis[24]. Isolated from *Tylophora indica* (Burn. L.) Mirr (Asclepiadaceae) leaves.

**41**

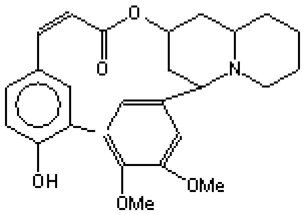

**Vertine**

Found in *Lagerstroemia faurei* (Lythraceae). Shows hypotensive and anti-inflammatory activity[8].

**42**

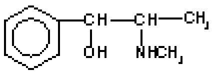

**Pseudoephedrine**

**43**

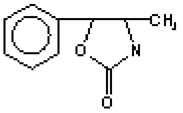

**Ephedroxane**

Since the traditional usage of an Oriental medicine "mao", consisting of Ephedra herbs (*Ephedra intermedia*, Ephedraceae) suggests that it may possess anti-inflammatory activity. The methanol extract was fractionated, resulting in the isolation of (+) pseudoephedrine[25] and a new alkaloid designated as ephedroxane. The anti-inflammatory effects were determined on carrageenan paw edema in mice, Whittle method and the fertile egg method[26].

**44**

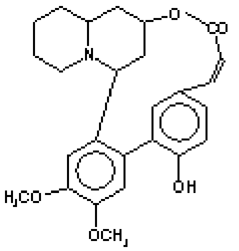

**Cryogenine**

**45**

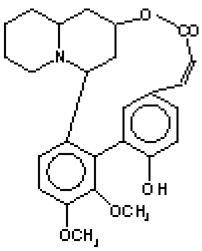

**Nesodine**

Two alkaloids, cryogenine and nesodine, isolated from *Heimia salcifolia* Link and Otto (Lythraceae) were shown to be 2.48 and 2.24 times as potent as aspirin in inhibiting the prostaglandin synthetase prepared from bovine seminal vesicles. Have been shown to exert anti-inflammatory activity in different systems[27].

**46**

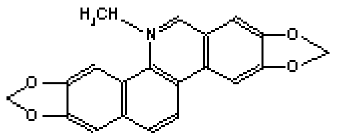

**Sanguinerine**

**47**

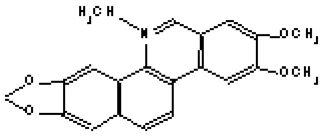

**Chelerythrine**

**48**

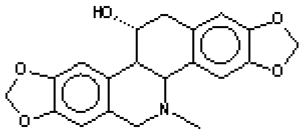

**Chelidonine**

The orange-colored latex of *Chelidonium majus* L. (Papaveraceae) contains high concentrations of benzophenanthridine alkaloids, specially of the main alkaloids chelidonine[28], chelerythrine, and sanguinarine[29]. The chelerythrine and sanguinarine are, to a great extent, responsible for anti-inflammatory and antimicrobial action. Recommended for medical use in the treatment of oral inflammatory processes. *C. majus* is a popular medicinal herb commonly widespread in Central Europe and represents a rich source of benzophenanthridine alkaloids.

**49**

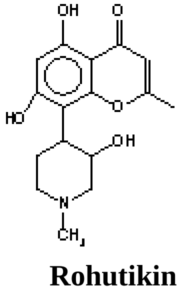

**Rohutikin**

**50**

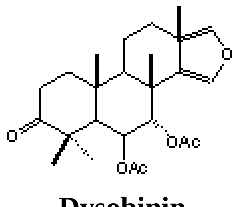

**Dysobinin**

Dysobinin, a new tetranortriterpene of the meliacin group showing general CNS-depressant action and mild anti-inflammatory activity, was isolated from the fruits of *Dysoxylum binectariferum* Hook. (Meliaceae)[30]. The alkaloid, rohutikin, isolated from the stem bark is an iperidinylbenzopyranone. Displayed anti-inflammatory activity in carrageenan-induced rat paw edema and inhibited the reverse passive Arthus reactions in rats (50.8% inhibition at 2.5 mg/kg, p.o.)[31].

## COUMARINS

**51**

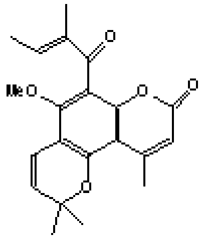

**Calophyllolide**

A nonsteroidal anti-inflammatory agent found to be effective in reducing the increased capillary permeability induced in mice by various chemical mediators involved in the inflammatory process viz., histamine, 5-hydroxytryptamine, and bradykinin[32]. Caused a decrease of 60.7% in the rat paw carrageenan edema at a dose of 40 mg/kg i.p. Its activity was compared with that of hydrocortisone, which at a dose of 10 mg/kg inhibited the edema by 44%. Showed the greatest anticonvulsant activity in rodents[33]. Isolated from Kernel of *Calophyllum mophyllum* Linn. (Guttiferae).

**52**

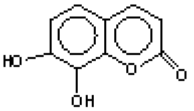

**Daphnetin**

Has displayed analgesic, sedative, and anti-inflammatory effects in a series of animals test[34]. Isolated from *Daphne giraldii* Nitsche (Thymelaeaceae) leaves.

**53**

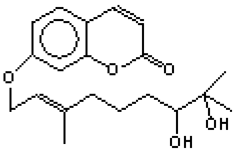

**Marmin**

Isolated from roots of *Aegle marmelos* Correa (Rutaceae). Showed anti-inflammatory effects in carrageenan-induced inflammation in rats[35].

**54**

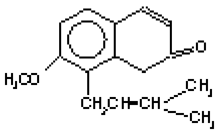

**Osthol**

The methanol extract of the root of *Angelica pubescens* Maxim. (Umbelliferae) was fractionated, and by following the inhibitory activities on rat hind edema induced by carrageenan and on writhing induced by acetic acid in mouse. The active principle was isolated and identified as osthol[36]. This plant has been used in traditional Chinese medicine as a remedy for arthritic disease, lumbago, edema, and the common cold.

**55**

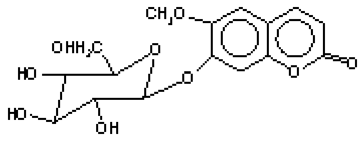

**Scopolin**

**56**

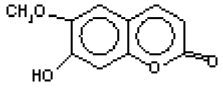

**Scopoletin**

Obtained from *Scopolia japonica* (Solanaceae). Demonstrate anti-inflammatory activity against acute albumin- or histamine-induced edema of hindpaws and acute subacute joint swelling caused by formaldehyde. Both compounds also exhibit analgesic effects[37].

**57**

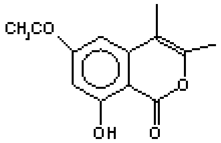

**Polygonolide**

A new isocoumarin. Inhibits the reversed passive Arthus reaction in rats via oral administration. Isolated from the methanol extract of the root of *Polygonum hydropiper* Linn. (Polygonaceae)[38].

**58**

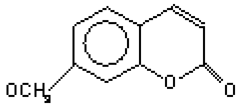

**Herniarin**

**59**

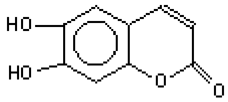

**Aesculetin**

Isolated from *Santolina oblongifolia* Boiss (Compositae). These coumarins showed considerable activity as inhibitors of eicosanoid release from ionophore-stimulated mouse peritoneal macrophages[39].

## FLAVONOIDS

**60**

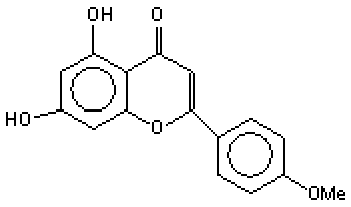

**Acacetin**

Found in *Robinia pseudocacia*. Is an anti-inflammatory, capillary protective, and spasmolytic agent[8].

**61**

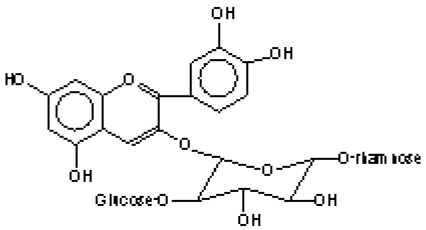

**Anthocyanin 1**

**62**

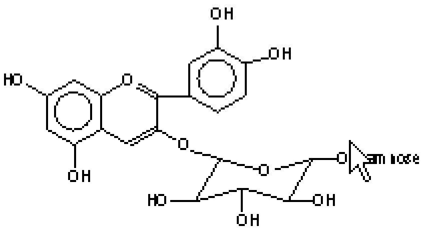

**Anthocyanin 2**

Occur in tart cherries. Exhibit antioxidant and anti-inflammatory activities comparable to commercial products[40].

**63**

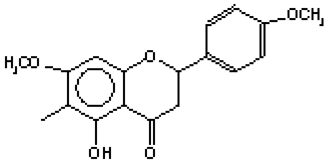

**Apigenin 7,4´-dimethyl ether**

Isolated from roots of *Rhus undulata* Jacq. (Anacardiaceae). Active on rat paw carrageenan edema with $ED_{25}$ = 75 mg/kg, while hydrocortisone phosphate and nobiletin were more potent: $ED_{25}$ = 13.5 and 20 mg/kg, respectively[41].

**64**

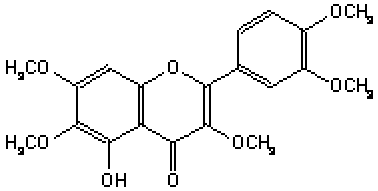

**Artemetin**

This 5-hydroxy-3,6,7,3´,4´-pentamethoxyflavone from the leaf of *Cordia verbenacea* (Boraginaceae) showed marked anti-inflammatory activity using various experimental models in rats. Also reduced the vascular permeability to intracutaneous histamine. Subacute toxicological experiments indicated a very low toxicity[42]. Extract for leaves of *C. verbenaceae* are used in Brazilian folk remedies as anti-inflammatory and cicatrizing agents[43].

**65**

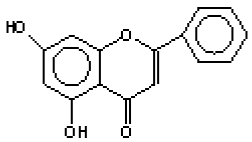

**Chrysine**

The flavonoid fraction of leaves of *Populus* spp. (Salicaceae) inhibits croton oil dermatitis in the mouse ear. This activity is mainly due to chrysin[41].

**66** **67**

Cinnamonol $D_1$ R= ---OH Cinnamonol $D_2$ R= —OH

Epicatechin, catechin, procyanidin $B_2$ and $B_4$, cinnamonol $D_1$ and $D_4$ isolated from aq. methanol extract of the bark of *Cinnamomun sieboldii* Meissn. (Lauraceae). Had potent inhibitory activity on the formation of granulation tissue through screening by the fertile egg methods[44]. The activity increased in the order of monomers, dimers, and trimers. Catechin is able to decrease carrageenan edema (ID = 240 mg/kg, i.p.), while at a lower dose (40 mg/kg s.c.) moderately inhibited rat paw edema induced by serotonin.

**68**

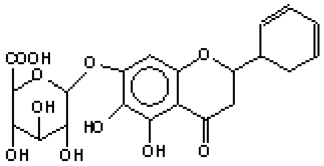

**Baicalin**

**69**

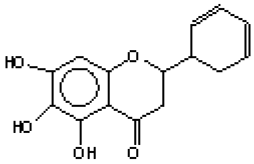

**Baicalein**

**70**

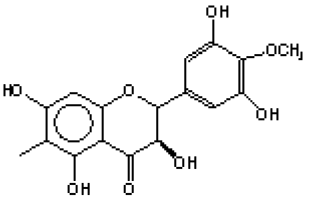

**Wogonin**

The main flavonoid components of leaves of *Scutellaria baicalensis* Georgi (Labiatae) — baicalin, baicalein, and wogonin — have been screened in comparison with three standard anti-inflammatory agents — phenylbutazone, indomethacin, and dexamethasone — for activity in various experimental models of inflammation. All of the test substances were found to inhibit an increase in vascular permeability in mice induced by acetic acid and to reduce acute paw edema in rats. They also suppressed the secondary lesion in developing adjuvant-induced arthritis in rats[45]. The mechanism of their anti-inflammatory action depends on inhibition of enzymes such as 12-lipoxygenase and 5-lipoxygenase. *S. baicalensis* (Ogon in Japanese) has been used for treatment of some types of dermatitis, diarrhea, and various inflammatory diseases as an antiphologistic and antipyretic in the traditional Chinese system of medicine.

**71**

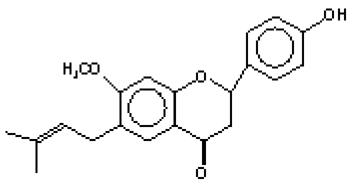

**Bavachinin**

A phenol compound isolated from the leaves of *Psoralea coryfolia* Linn (Leguminosae) has shown anti-inflammatory activity[46].

**72**

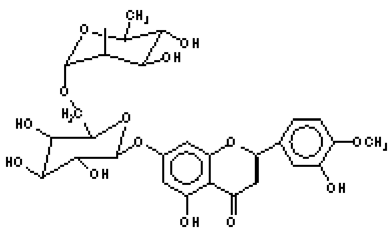

**Diosmin**

Exhibited anti-inflammatory activity and increased skin capillary resistance[47]. Isolated from leaves of *Diosma crenulata* (Rutaceae).

**73**

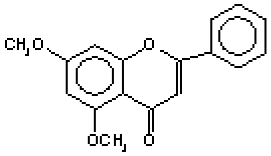

**5-7-Dimethoxyflavone**

Isolated from black rhizomes of the *Boesenbergia pandurata* Robxb. Zingiberaceae, the anti-inflammatory activity has been assessed. Found to possess comparable effects to aspirin on the rat edema model, and showed no inhibition on cotton pellet–induced granuloma formation. On the rat pleurisy model, it exhibited an antiexudative effect, interfered with leukocyte migration, and markedly inhibited prostaglandin biosynthesis[48].

**74**

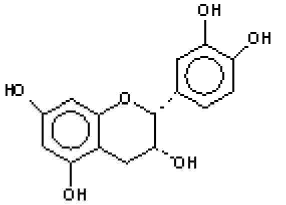

**Epicatechin**

A catechin with significant anti-inflammatory activity isolated from the seed of the plant *Anacardium occidentale* Linn (Anacardiaceae)[49].

**75**

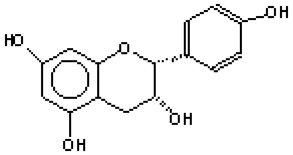

**(-)Epiafzelechin**

An inhibitor of cyclo-oxygenase (COX-1) activity of prostaglandin $H_2$ synthase. Isolated from aerial parts of *Celastrus orbiculatus* (Celastraceae). An Oriental folk medicine for rheumatoid arthritis[50].

**76**

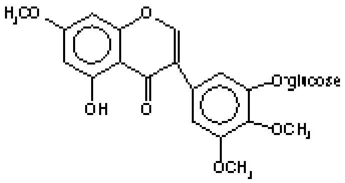

**Flavan-3-ol, (+)-catechin**

Showed suppression of carrageenan-induced rat paw edema as well as TPA-induced rat ear edema (tetradecanoylphorbol acetate)[51]. Isolated from the bark of *Ceiba pentandra* L. Gaether.

**77**

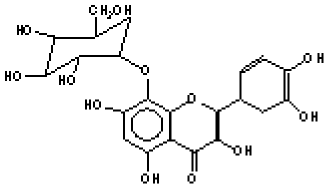

**Gosspyn**

Glycoside (gossypetin-7-glucoside) from the flowers of *Hibiscus vitifolius* L. (Malvaceae). Inhibits the carrageenan rats paw edema[27].

**78**

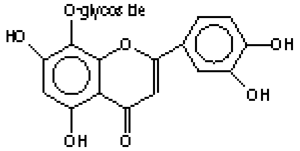

**Hypolaetin-8-glucoside**

Had an interesting anti-inflammatory profile. This glucoside was more potent than phenylbutazone in suppressing the acute phase of the adjuvant-carrageenan-induced inflammation, but was less effective in the prolonged phase[52,53]. Isolated from leaves of *Sideritis mugronensis* (Labiatae).

**79**

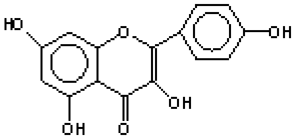

**Kaemferol 3,5,7,4´tetrahydroxyflavone**

Isolated from flowers of *Aesculus hippocastanum* (Hippocastanaceae). Shows anti-inflammatory, antibacterial, and mutagenic activities[41].

**80**

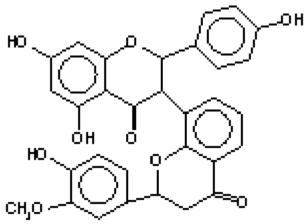

**Kolaflavanone**

Isolated from the rind of the fruit of *Garcinia kola* (Guttiferae). Inhibited primary and secondary responses of adjuvant induced arthritis in rats[54].

**81**

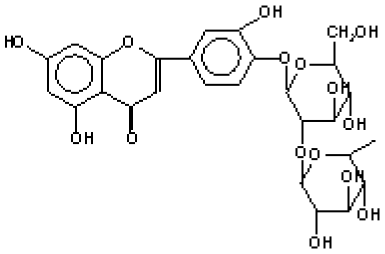

**Luteolin-4´-neohesperidoside**

This flavonoid glycoside was isolated from *Caralluma attenuata* (Asclepiadaceae). Has anti-inflammatory activity[55].

**82**

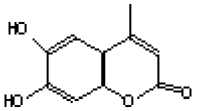

**Methyl 4-esculetol**

Showed activity against initial symptoms of inflammation carrageenan edema, dextran edema, histamine wheal, and peritoneal permeability[56]. Isolated from leaves of *Ruscus aculeatus* L. (Liliaceae).

**83**

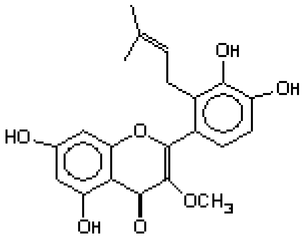

**Podaverine A**

**84**

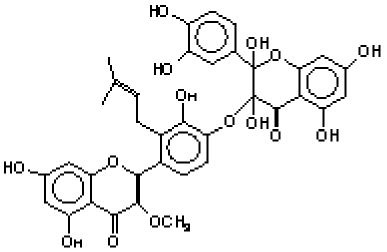

**Podoverine B**

Isolated from cell culture of leaves of *Podophyllum versipelle* Hance (Berberidaceae). Showed inhibitory activities in the mouse macrophage chemiluminescense (CL) assay and are responsible for the activity of the corresponding callus culture. Preliminary *in vivo* studies indicate that the compounds have anti- inflammatory activity. These inhibitory activities are comparable to those of the antioxidant compound quercetin and nordihydroguaiaretic acid[57].

**85**

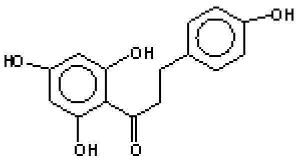

**Phloretin**

Occurs as the 2´-glucoside, specially in Rosaceae, Ericaceae, and Symplocaceae. Showed anti-inflammatory activity[58].

**86**

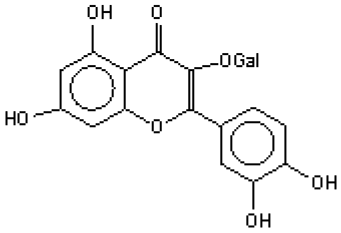

**Quercetin-3-O-galactoside**

Exhibited anti-inflammatory activity comparable to phenylbutazone in carrageenan-induced rat paw edema model[59]. Isolated from leaves of *Physalis minima* Linn (Solanaceae).

**87**

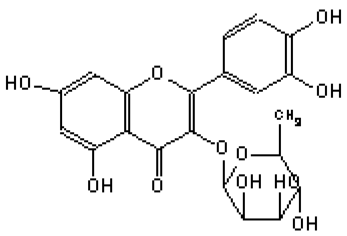

**Quercetin 3O-rhamnoside**

**88**

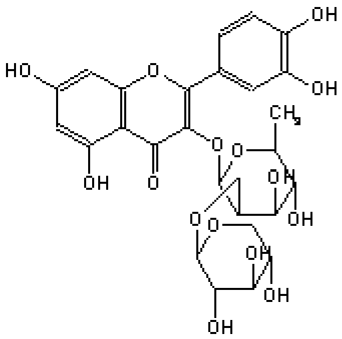

**Quercetin 3O-xylosyl(1→2) rhamnoside**

Two anti-inflammatory principles — quercetin 3O-xylosyl(1→2) rhamnoside and quercetin 3O-rhamnoside — isolated from the methanol extract of the leaves of *Erythrospermum monticolum.* Active against the acute inflammation in mice induced by TPA (12-O-tetradecanoylphorbol acetate) producing significant reductions in edema. Their activity is in range of the well-established reference drug, indomethacin[60]. Quercetin, quercetin 3-rhamnoglucoside and 3-galactoside, rutin has been claimed to be the anti-inflammatory principle of *E. monticolum* (Flacourtiaceae), *Wrighitia tinctoria* (Apocynaceae), and *Delonix elata* Gamble (Leguminosea) flowers active on carrageenan edema.

**89**

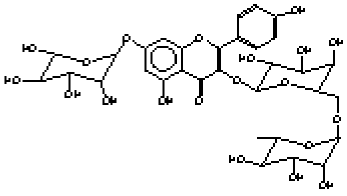

**Robinin**

The kaempferol glycoside robinin isolated from *Robinia pseudacacia* Borja (Leguminosae) leaves inhibits the exudative and proliferative phases of the cotton pellet granuloma (50 mg/kg/day p.o. in 7 days)[56]. Used as an anti-inflammatory and antirheumatic agent in Spain's folk medicine.

**90**

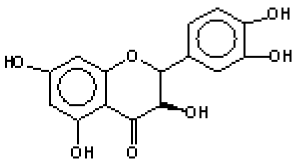

**Taxifolin**

**91**

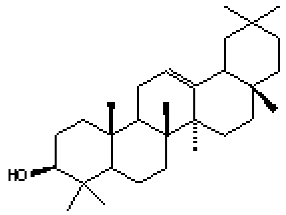

**α-Amyrin**

Taxifolin and α-amyrin from the seed of *Cordia obliqua* Wild. (Boraginaceae) showed significant anti-inflammatory activity[41]. The polar fraction of the plant extract *Cedrus deodara* (RoxB) Loud. (Pinaceae) was found to exhibit anti-inflammatory activity in carrageenan-induced edema in mice and its chemical examination has resulted in the isolation of dihydroflavanonols[61].

**92**

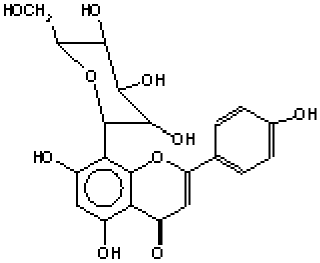

**Vitexin**

(8-β-D-glucopyranosyl-apigenin) (VT). Isolated from the roots and flowers of *Arnebia nobilis, A. gullata, A. benthamii, A. hispidissima* DC. (Boraginaceae), and *Ochrocarpus longifollus* L. (Guttiferae). Exhibited potent hypotensive, anti-inflammatory, and antispasmodic properties. An anti-inflammatory effect probably related to its antihistamine, antibradykinin, and antiserotonin properties[62].

**93**

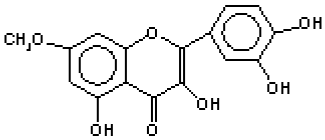

**Xanthorhamnin**

Isolated from the seeds of *Rhamnus infectoria* (Rhamnaceae). Has been patented as an anti-inflammatory agent and recommended for the treatment of rheumatoid arthritis and for use in ophthalmology[63].

**94**

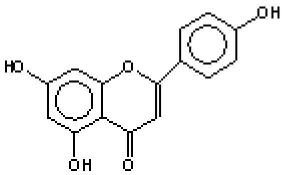

**Apigenin**

**95**

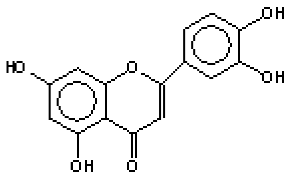

**Luteolin**

Apigenin and luteolin showed a potency similar to indomethacin ($ID_{50}$ = 29.8 and 38.4 μ per ear, respectively). 18 h after the treatment, only luteolin maintained its effects on edema development but the three compounds decreased leukocyte infiltration[64]. Isolated from leaves of *Chamomilla recutita* L. (Compositae).

**96**

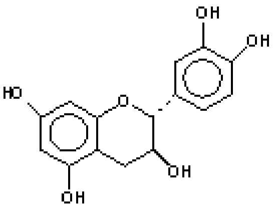

**97**

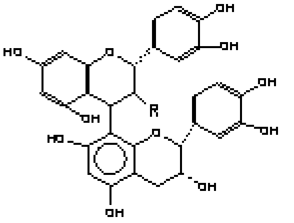

**98**

Procyanidin $B_4$ R= —OH

**Catechin** **Procyanidin $B_2$ R= ---OH**

Isolated from *Cinnamomun sieboldii*[44].

**99**

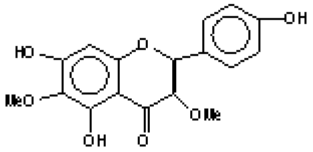

**100**

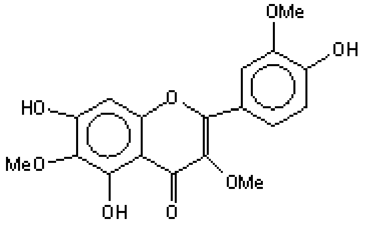

**6-Hhydroxykaempferol,3,6-dimethyl ether** **Quercetagenin 3,6,3´-trimethyl ether**

6-Hydroxykaemperol-3,6-dimethyl ether has an equivalent profile of enzyme inhibitory activity to santin in both assay (cyclo-oxygenase and 5-lipoxygenase pathways), but is less potent. Quercetagetin 3,6,3´-trimethyl ether shows preferential activity against cyclo-oxygenase with much less activity in inhibiting 5-lipoxygenase[65]. Isolated from *Tanacetum parthenium* and *T. vulgare* (Compositae)[66].

**101**

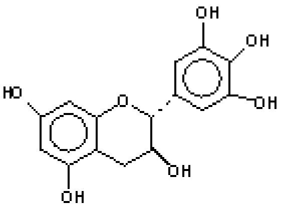

**102**

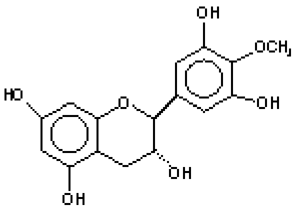

**103**

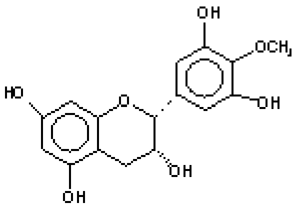

**Gallocatechin** **4´-O-methyl-ent-gallocatechin** **Ouratea-catechin**

All catechin isolated from *Atuna racemosa* (Chrysobalanaceae) and *Syzygium corynocarpum* (Myrtaceae) exhibited strong to medium inhibition of COX-1 catalysed prostaglandin biosynthesis *in vitro*[67].

**104**

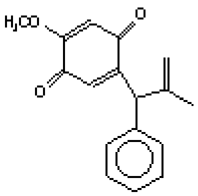

**(S)-4-methoxydalbergione**

**105**

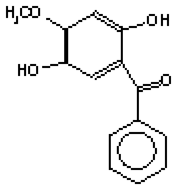

**Cearoin**

**106**

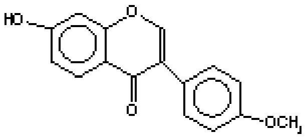

**Koparin**

**107**

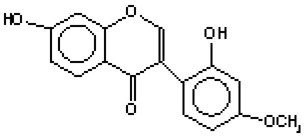

**Xenognosin**

**108**

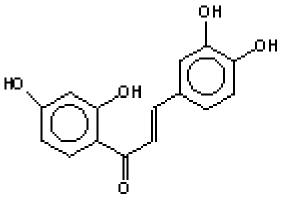

**Butein**

**109**

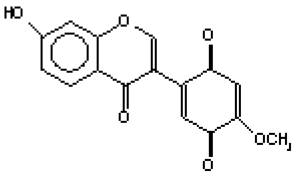

**Bowdichione**

**110**

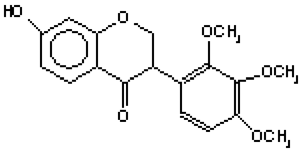

**3-O-methylviolanone**

Found in heartwood of *Dalbergia odorifera* T. Chen. (Leguminosae). A traditional Chinese medicine known as jiangxiang. Has been used to treat blood disorders, ischemia, swelling, necrosis, and rheumatic pain. Showed significant anti-inflammatory activity[68].

**111**

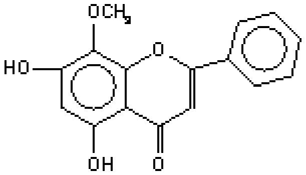

**Wogonin**

Found in the root of *Scutellaria baicalensis* Georgi (Labiatae). Inhibited IL-1β-induced synthesis of $PGE_2$ and $LTB_4$ considerably. In addition, these compounds exerted a moderate inhibition of collagenolytic activity and the cellular activity of fibroblast was increased remarkably[69].

**112**

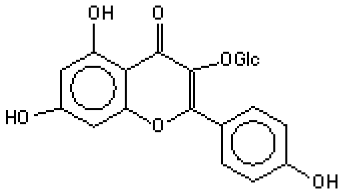

**Kaempferol-3-O-sophoroside (K3S)**

A polyphenolic constituent isolated of the alcoholic extract of the leaves of *Cassia alata* L. (Leguminosae). Has been investigated for anti-inflammatory activity and the results compared with phenylbutazone. Showed a significant anti-inflammatory effect in rats in carrageenan and induced hind paw edema test[70]. *C. alata* is used in India for skin diseases like eczema, pruritis, and itching.

## MISCELLANEOUS COMPOUNDS

**113**

$CH_2{=}CHCH_2S(O)CH_2CH{=}CHSSCH_2CH{=}CH_2$

**Ajoene**

Isolated from garlic (*Alium sativum*, Liliaceae). Showed potent inhibition of platelet aggregation[8].

**114**

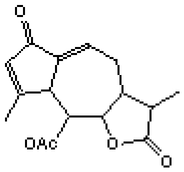

**Arnicolide**

Inhibited the carrageenan- and formaldehyde-induced inflammation. Isolated from *Arnica montana* (Compositae) leaves[71].

**115**

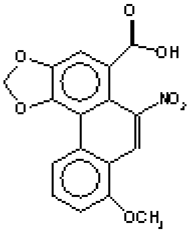

**Aristolich acid**

Inhibited the carrageenan- and formaldehyde-induced inflammation[72]. Isolated from leaves of *Aristolochia clematis* (Aristolochiaceae).

**116**

**Bromelain**

A basic glycoprotein isolated from the fruit pineapple (*Ananas comosus*, Bromiliaceae). This proteolythic enzyme is used medicinally as an anti-inflammatory agent in soft tissue edema and injury[73].

**117**

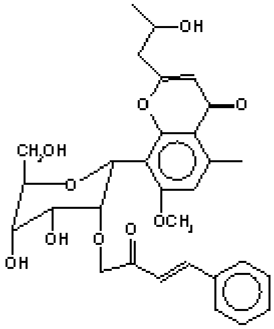

**8[C-β-D-[2-O-(E)-cinamoylglu-copyranosyl]-2-[®-2-hydroxypropyl]-7-methoxy-5-methylchromone**

Found in *Aloe barbadensis* Mill (Liliaceae). At a dose of 200 μ per mouse ear exhibited topical anti-inflammatory activity equivalent to 200 μ per ear of hydrocortisone[74].

**118**

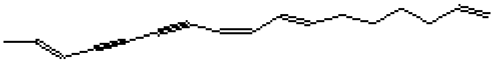

**Heptadeca-2E,8E,10E,16-tetraene-4,6-dyne**

**119**

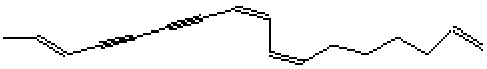

**Heptadeca-2E,8Z,10E,16-tetraene-4,6-dyne**

**120**

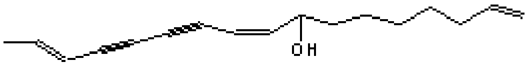

**Heptadeca-2E,8E,16-triene-4,6-dyne-10-ol**

Found in *Bidens campylotheca* Schultz Bip (Compositae). This plant is a traditional remedy in Hawaiian folk medicine for treating general weakness of the body, throat and stomach disorders, and of asthma. Showed significant *in vitro* inhibition of cyclo-oxygenase (CO) and 5-lipoxygenase (5-LO)[75].

**121**

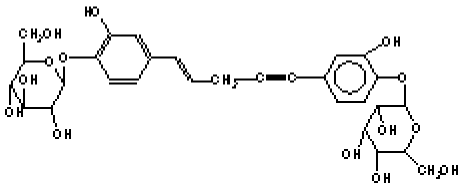

**Hypoxoside**

Has a pronounced anti-inflammatory (rat paw edema) and fairly good analgesic effect[76]. Isolated from *Hypoxis obtusa* (Hypoxidaceae) rhizomes.

**122**

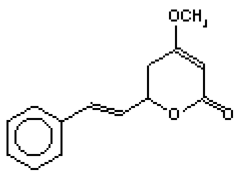

**Kawain**

Occurs in the rhizomes of *Piper methysticum* (Piperaceae). Has anti-inflammatory activity[77].

**123**

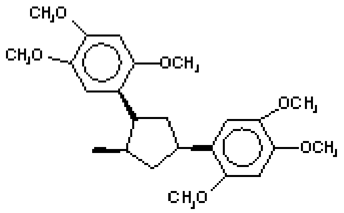

**Magnosalicin**

Obtained from the dried buds of *Magnolia salicifolia* (Magnoliaceae), significantly inhibited granuloma tissue formation. The inhibitory effect was particularly strong, being nearly half of hydrocortisone acetate when administered orally[78].

**124**

$CH_2(OR)CH{=}(OH)\ CH_2OH$

**Monoglycerides**

The monoglycerides $CH_2(OR)CH{=}(OH)CH_2OH$ (R=palmitoyl, linoleoyl, linolenoyl, or oleoyl) are isolated from *Aconitum chinense* (Ranuncolaceae) as analgesic and anti-inflammatories from *A. chinense*[79].

**125**

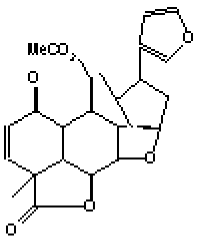

**Nimboline**

Has pronounced anti-inflammatory (rat paw edema) and fairly good antipyretic effect (pyrogen-induced hyperpyrexia in rabbits). The acute oral toxicity in mice showed a very low range of approximately 13 g/kg body weight[80,81]. Isolated from leaves of *Rachta indica* (Meliaceae).

**126**

**Pedilanthain**

A new protease isolated from the latex of *Pedilanthus tithymaloides* Poit Euphorbiaceae and subjected to anti-inflammatory screening. More potent than phenylbutazone. Exhibits its maximum activity at 2nd hour of its oral administration[82].

**127**

**Polysaccharide**

Produced by *Serratia piscatorum*. Exhibited anti-inflammatory activity which was completely lost when hydrolyzed into small molecules[83].

**128**

**Polysaccharide**

Isolated from *Echinocea angustifolia* DC. (Compositae). Enhance phgocytosis *in vivo* an dexhibit anti-inflammatory activity[84].

**129**

**Polysaccharide $M_t$´S $1.5 \times 10^5$**

**130**

**Polysaccharide $M_t$´S $3.6 \times 10^4$**

**131**

**Polysaccharide $M_t$´S $1.5 \times 10^4$**

Three polysaccharide glucans were isolated from dried roots of *Periandra mediterranea* Taubert (Leguminosae). Widely used in Brazilian ethnomedicine as a cough suppressant. Highly immunostimulatory[85] and anti-inflammatory properties.

**132**

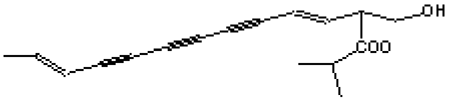

**Safynol-2-O-isobutyrate**

**133**

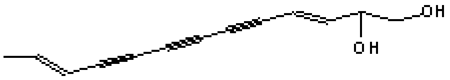

**Safynol**

Isolated from *Bidens campylotheca* Schultz Bip (Compositae)[75].

**134**

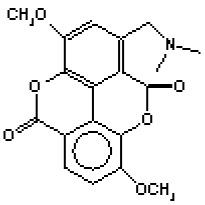

**Taspine**

*Croton lechleri* L. (Anacardaceae), commonly called “Sangre de grado”, is a tree of the upper Amazon valley of Peru. The bark, when slashed, produces a red viscous sap which is used for the treatment of rheumatism. Shown to elicit anti-inflammatory activity in different models[86].

**135**

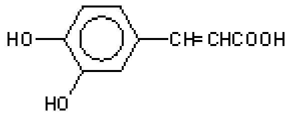

**Caffeic acid**

**136**

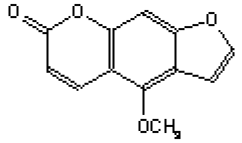

**Bergapten**

**137**

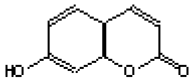

**Umbelliferone**

**138**

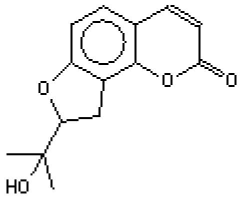

**Columbianadin**

Caffeic acid, bergapten, columbianadin, and umbelliferone isolated from *Angelica pubescens* Maxim (Umbelliferae). Significantly demonstrated anti-inflammatory and analgesic activities at 10 mg/kg in the test: hindpaw, peritoneal vascular permeability, skin window, hot plate, and formalin[87]. The anti-inflammatory and analgesic activities of osthole were reported by Kosuge et al. in 1985[88] from the same plant. Caffeic acid - widespread occurrence in green and roasted coffee beans *Coffea arabica*, (Rubiaceae), the root bark of *Chinchona cuprea*, Rubiaceae), and in *Conium maculatum* (Umbelliferae). Also occurs in herbaceous plants such as *Digitalis purpurea* (Scrophulariaceae), the leaves and flowers of *Papaver sommiferum* (Papaveraceae), the roots of *Taraxacum officinale*, and the flowers of *Anthemis nobilis* (Compositae) and *Achillea millefolium* (Compositae)[89]. Umbelliferone - widespread ocurrence in the resin of various Umbelliferae: *Ferula, Apium, Pimpinella,* and *Heracleum* spp. Used in sunscreen lotions.

## MONOTERPENOIDS, DITERPENOIDS, AND SESQUITERPENOIDS

**139**

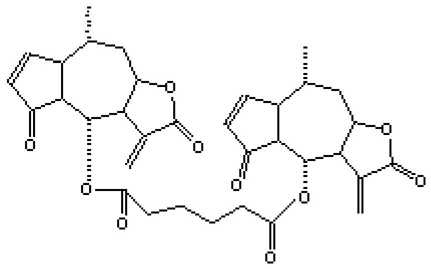

**Bis[helenalinyl]adipate**

Isolated from *Schizogyne sericea* (Compositae) leaves. Showed anti-inflammatory activity in rats at 50 mg/kg, i.p. dose[90].

**140**

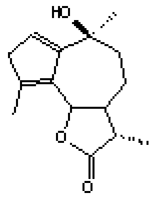

**Artabsin**

Found in wormwood of *Artemisia absinthium* (Compositae). Has anti-inflammatory activity[91].

**141**

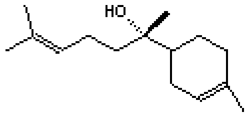

**α-Bisabolol**

Isolated from chamomile oil from *Matricaria chamomilla* (Compositae). Has anti-inflammatory activity, but is less toxic than guaiazulene, which has a similar action[92].

**142**

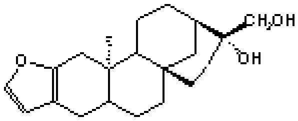

**Cafestol**

The main constituent of the unsaponifiable portion of coffee bean oil. From *Coffea* spp. (Rubiaceae). Showed anti-inflammatory activity against carrageenan-induced edema in rats[93].

**143**

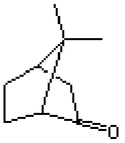

**Camphor**

Constituent of *Matricaria parthenium* (Compuestas). Has a pronounced anti-inflammatory activity in rat paw edema[94].

**144**

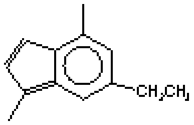

**Chamazuleno**

A blue oil produced during steam distillation of chamomile with anti-inflammatory and antipyretic activities[92]. Isolated from leaves of *Matricaria chamomilla* (Compositae).

**145**

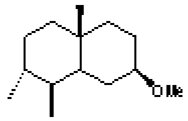

**Chrysanthemol**

A new compound isolated from *Crysanthemun indicum* L. (Compositae) flowers that showed strong anti-inflammatory activity in mice[95].

**146**

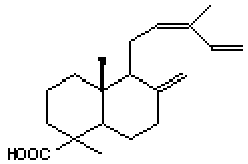

**Cis-communic acid**

Found in *Cryptomeria japonica* Don (Taxodiaceae). Has anti-inflammatory activity. The activity testing was done using the carrageenan-induced paw edema (CPE) method in rats[96]. The leaves of *C. japonica* have been used traditionally in Chinese medicine for the treatment of eczema, eruption, and swelling injury by topical application. Cis-communic acid showed anti-inflammatory effect when applied topically to rats and an inhibitory effect on histamine-induced ileum contraction[86].

**147**

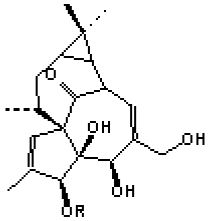

R=COCH=CHCH=CH-$(CH_2)_4$-$CH_3$

**3-O-1 (2´É,4´Z-decadienoyl)-hydroxyingenol**

**148**

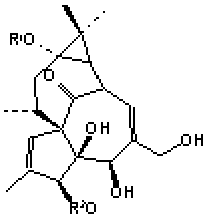

$R^1$= $H_3C$-$(CH_2)_{10}$-CO

$R^2$=$(H_3C)_2$CHCH(CH3)CO

**3-O-(2,3-dimethylbutyryl)-13-O-n-dodecanoyl-13-hydroxyingenol**

Immune complex binding to macrophages was enhanced by treatment with a *Euphorbia kansui* Liou (Euphorbiaceae) extract. Systematic fractionation of the extract led to the characterization of 3-O-1(2´É,4´Z-decadienoyl)-hydroxyingenol and 3-O-(2,3-dimethylbutyryl)-13-O-n-dodecanoyl-13-hydroxyingenol as the active principles. Immune complex binding to macrophages by the action of these compounds increased in a dose-dependent manner[97].

**149**

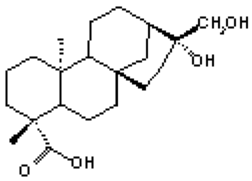

**Diterpenoid SP-II**

Found in *Sigesbeckia pubescens* (Compositae) together with several other related diterpenoids. Showed anti-inflammatory activity and is a powerful antihypertensive[93].

**150**

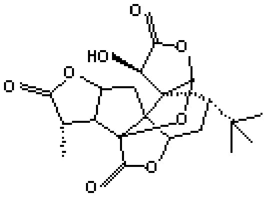

**Ginkgolide A**

Found in the root bark and leaves of maidenhair tree, *Ginkgo biloba* (Ginkgoaceae). Used medicinally to treat allergic inflammation and asthma[91].

**151**

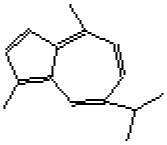

**Guaiazulene**

A blue oil produced during steam distillation of chamomile with anti-inflammatory and antipyretic activities[92]. Extracted from *Matricaria chamomilla* (Compositae).

**152**

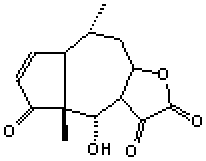

**Helenalin**

Sesquiterpene lactones have previously been shown to possess anti-inflammatory and antiarthritic activities in rodents. The α-methylene-γ-lactone moiety of this chemical class is required for activity as well as the β-unsaturated cyclopentenone ring. The helenalin showed significant inhibitory activity against carrageenan-induced edema[90]. Isolated from *Helenium autumale, H. anaphalis, H. balduinea,* and *H. gaillardia* (Compositae).

**153**

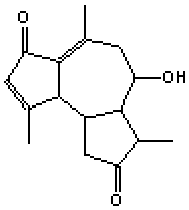

**Hydroxyachillin**

Found in the aerial parts of *Tanacetum microphyllum* (Compositae). Used in the Iberian peninsula since ancient times in Spanish traditional medicine as an anti-inflammatory and antirheumatic agent, and for its beneficial effects on the digestive tract. Showed a marked anti-inflammatory activity in carrageenan-induced paw edema in mice[98].

**154**

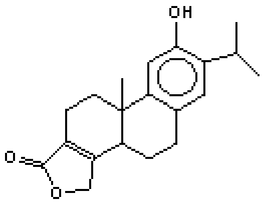

**Isotriptophenolide**

An abietane diterpene from the perennial herb *Tripterygium wilforddi* Hookf. (Celastraceae). Shown to possess anti-inflammatory activity[99]. Has gastric cryoprotective effect[100].

**155**

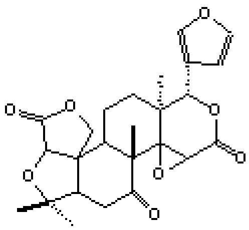

**Limonin**

Possesses an antinociceptive effect accompanied by an anti-inflammatory action. Isolated from *Evodia rutaecarpa* var. Bodinieri (Rutaceae)[101].

**156**

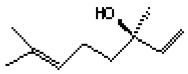

**Linalool**

The main constituent of oil coriander from *Coriandrum sativum* (Umbelliferae) and *Thymus quinquicostatus*. Exhibits anti-inflammatory action[102].

**157**

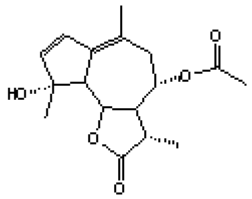

**Matricin**

The guaianolide was isolated from chamomile, *Matricaria chamomilla* (Compositae). Precursor of chamazulene, which is anti-inflammatory[91].

**158**

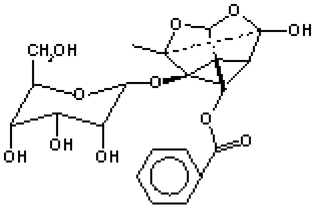

**Paeoniflorin**

Found in *Paeonia lactiflora* (Paeoniaceae). Has anti-inflammatory activity[103].

**159**

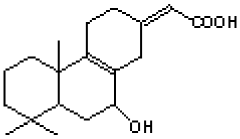

**Rhinocerotinoic acid**

The structure was determined as 7-oxolabda-8-13-dien-15(E)-oic acid by means of X-ray. Showed anti-inflammatory activity in rats[104]. Isolated from leaves of *Elytropappus rhinocerotis*.

**160**

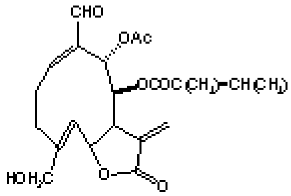

**Sesquiterpene**

Isolated from *Siegesbeckia pubescens* (Asteraceae) leaves at 100 mg/kg p.o. Inhibited inflammation by 44.9% in the carrageenan edema test using male Wistar rats[105].

**161**

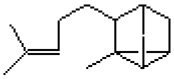

**β-Santalen**

Anti-inflammatory activity of the petroleum ether extract of *Conzya canadensis* L. Cronq (Asteraceae) has been reported. The fractionation of the extract led to the isolation of five sesquiterpenes: β-santalen, β-himachalen, cuparene, α-curcumene, γ-cardinene with anti-inflammatory activity[106].

**162**

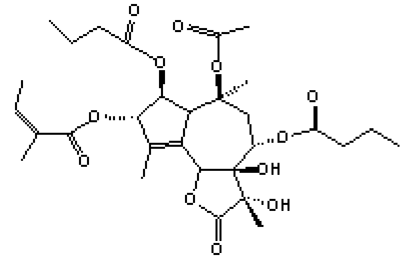

**Thapsigargin**

Found in *Thapsia garganica* (Umbelliferae). Potent activator of cells involved in anti-inflammatory response[91].

**163**

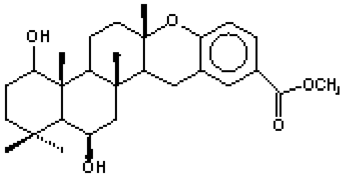

**Tolypodiol**

The diterpenoid has been isolated from the terrestrial cyanobacterium, *Tolypothix nodosa* (HT-58-2). Shows potent anti-inflammatory activity in the mouse ear edema assay[107].

**164**

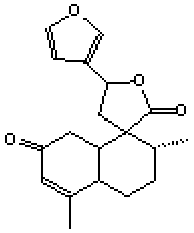

**Trans-dehydrocrotonin**

Occurs in *Croton cajucara* Benth. (Euphorbiaceae). Widely known in traditional phytotherapy for the treatment of diabetes, diarrhea, gastrointestinal disorders, and liver diseases. Also indicated for controlling high cholesterol levels. Its vernacular name is sacasa and it occurs widely in the Amazon region (Brazil). Produced a significant inhibition of carrageenan-induced paw edema and cotton pellet granuloma in rats. It also inhibited the writhing in mice induced by acetic acid[108].

**165**

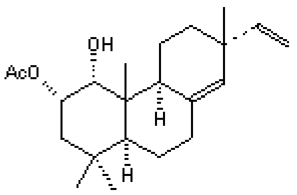

**2α-Acetoxysandaracopimaradien-1α-ol**

**166**

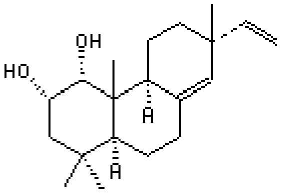

**Sandaracopimaradien1α-2α-diol**

The two diterpenes isolated from *Kaempferia pulchra* Ridl. (Zingiberaceae) have been assayed for topical anti-inflammatory activity in the model of TPA-induced ear edema in rats[109].

**167**

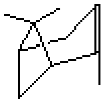

**α Pinene**

**168**

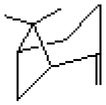

**β Pinene**

**169**

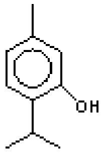

**Thymol**

**170**

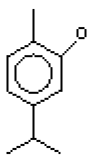

**Carvacrol**

The essential oil of *Bupleurum fructicosum* L. (Umbelliferae) has shown significant anti-inflammatory activity in carrageenan-induced rat paw edema model and the activity is attributed in part to the major components, α and β pinene. It has also been shown that thymol and carvacrol, although present in extremely low levels, potentiate the pharmacodynamic action[106].

**171**

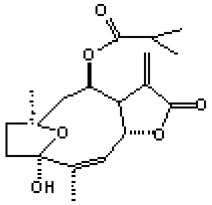

**Tirotundin**

**172**

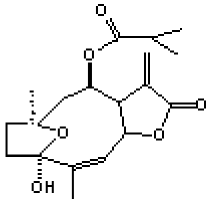

**Diversifolin**

Sesquiterpene lactones from *Tithonia diversifolia* (Hemsl) A. Gray (Asteraceae). In Central America, leaf extracts from this plant are used for the treatment of hematomas and wounds. Inhibit cyclo-oxygenase1, phospholipase $A_2$, or the transcription factor NF-KB[110].

**173**

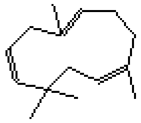

**β-Humulene**

**174**

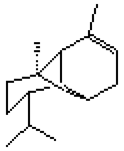

**α-Copaene**

**175**

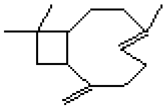

**β-Caryophyllene**

*Copaiba oleoresina* from Brazilian *Copaifera* spp. (Leguminosae). Has a widespread use in popular medicine as an anti-inflammatory agent. The oral administration of the oleoresin inhibited the development of carrageenan-induced edema in rats in dose-dependent manner. The oleoresin showed the presence of copalic acid and sesquiterpenes like β-bisabolene, β-caryophyllene, β-cubelene, aromandrene, β-humulene, and α-copaene[106,111].

**176**

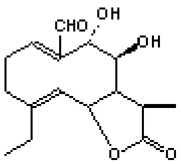

**8β-Hydroxy-9α-methacryloyloxy-14-oxo-acanthospermolide**

**177**

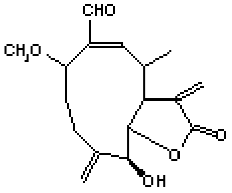

**1β-Methoxymiller-9Z-enolide**

**178**

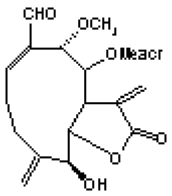

**9α-Methoxymiller-1-(10)-Z-enolide**

**179**

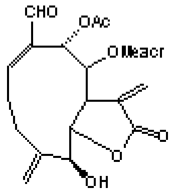

**9α-Acetoxymiller-1-(10)-Z-enolide**

**180**

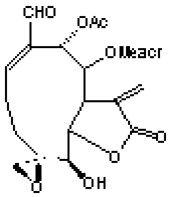

**9α-Acetoxy-4β,15-epoxymiller-1-(10)-Z-enolide**

In Central America, aerial parts of the *Milleria quinqueflora* (Asteraceae) are used in traditional medicine as a remedy for skin infections. All sesquiterpene lactones have anti-inflammatory activity using transcription factor NF-kB as molecular target. This is involved in the synthesis of inflammatory mediator, such as cytokines and chemokines. NF-kB -DNA binding was inhibited at micromolar concentrations by all compounds[112].

**181**

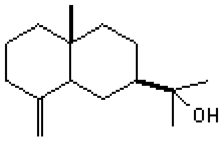

**β-Eudesmol**

**182**

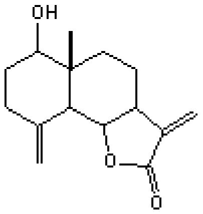

**Reynosin**

**183**

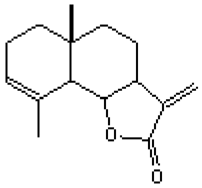

**α-Cyclocostunolide**

**184**

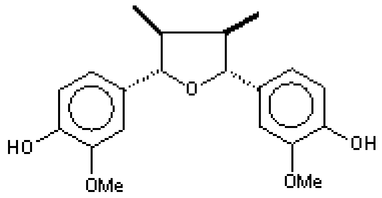

**Nectandrin-B**

**185**

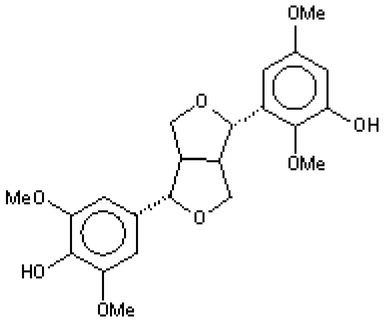

**Syringaresinol**

Found in *Tsoongiodendron odorum* and *Manglietiastrum sinicum* (Magnoliaceae). Displayed considerable inhibition against platelet aggregation induced by arachidonic acid (AA), adenosine diphosphate (ADP), and revealed inhibition activity against PAF-induced platelet aggregation[113].

## PHENOLICS COMPOUNDS

**186**

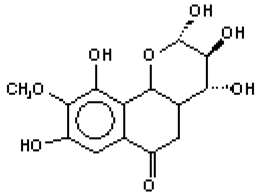

**Bergenin**

Occurs in *Peltophorum pteracarpum*, *P. inerme*, *Caesalpinia digyna* (Leguminosae), *Ardisia hortorum*, *Astilbe macroflora*, *Bergenia crassifolia* (Saxifragaxeae), *Corylopsis* spp. (Hamamelidaceae), and *Humuria balsamifera* (Humiriaceae)[77]. Found to be equipotent to phenylbutazone in rats against carrageenan-induced edema.

**187**

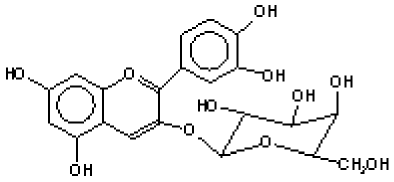

**Cyanidin-3-O-galactoside**

Has anti-inflammatory activity and is used in the prevention of capillary fragility[114]. Isolated from leaves of *Fagus sylvatica* (Fagaceae).

**188**

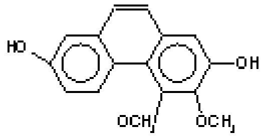

**2,7-Dihydroxy-3,4-methoxyphenantreno**

Showed inhibitory effect on contraction of guinea pig ileum induced histamine, while the topical application showed inhibition of edema in carrageenan-induced rat paw edema model[46]. Isolated from leaves of *Catastum barbatum* Lindl. (Orchidaceae).

**189**

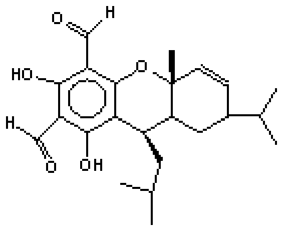

**Euglobal-1[a]**

Showed granulation inhibitory activity. One of a series of related compounds from *Eucalyptus globulus* (Myrtaceae) having the same activity[115].

**190**

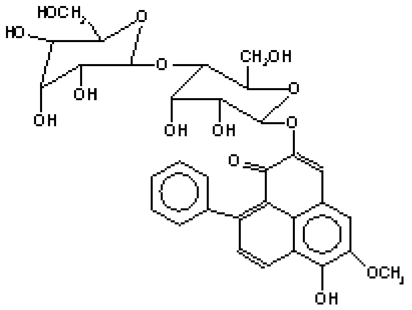

**Haemocorin**

Found in *Haemodorum coxymbosum* (Haemodoraceae). This aglycone showed anti-inflammatory activity[115].

**191**

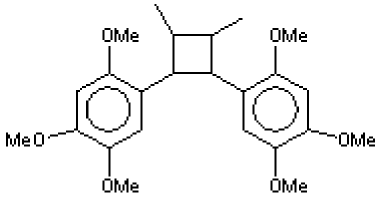

**Magnosalin**

**192**

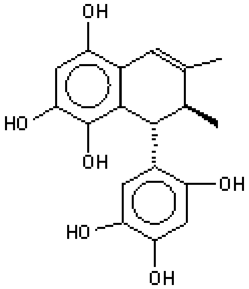

**Magnoshinin**

Found in *Magnolia salicifolia* (Magnolaceae). Possess inhibitory effects on granuloma formation[116].

**193**

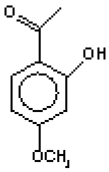

**Paenol**

Obtained from the traditional Chinese drug, Mu-San-Pi. Has bacteriostatic, anti-inflammatory, and CNS depressive effects[117]. Isolated from *Paeonia suffruticosa* Andr. (Paeoniaceae) roots.

**194**

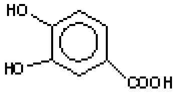

**Protocatechuic acid**

Found in *Erica australis* (Ericaceae). Has anti-inflammatory activity[46].

**195**

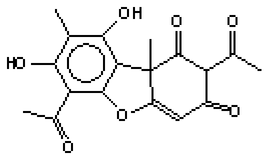

**Usnic acid**

**196**

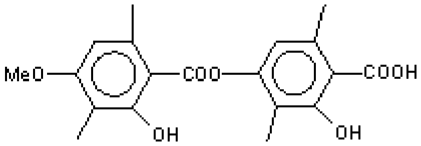

**Diffractaic acid**

The active principle usnic and diffractaic acid isolated from *Usnea diffracta* Vain (Usneaceae) exhibited anti-inflammatory activity when administered a dose of 50 mg/kg. Significantly inhibited the granulation tissue formation induced by subcutaneous implantation of cotton pellet[24].

**197**

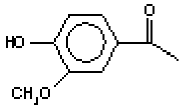

**Vanillic acid**

Occurs in *Fagara* spp. (Rutaceae), *Alnus japonica* (Betulaceae), *Eleagnus pungens* (Eleagnaceae), *Erica australis* (Ericaceae), *Gossypium mexicanum* (Malvaceae), *Melia azedarach* (Meliaceae), *Panax ginseng* (Araliaceae), *Patecoma koraiensis* (Bignoniaceae), *Pterocarpus santalinus*, *Rosa canina* (Rosaceae), *Picrorhiza kurroa* (Scrophulariaceae). and *Trachelospermum asiaticum* (Umbelliferae). The vanillic acid *in vitro* test indicated anti-inflammatory activity[46].

**198**

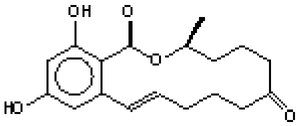

**Zearalenone**

A mycotoxin of fungus *Gibberella zeae*, which infects maize. Caused significant dose-dependant inhibition of carrageenan-induced paw edema[115].

**199**

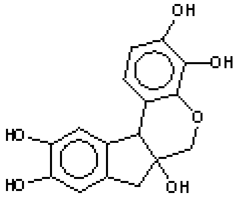

**Hematoxylin**

**200**

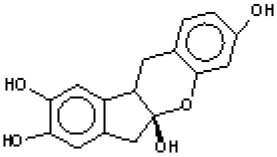

**Brazilin**

Brazilin together with hematoxylin, a constituent of *Haematoxylon campechianum* Linn. and *Caeselpinia sappan* Linné (Leguminosae) wood, have been subjected to various anti-inflammatory assays. Both substances proved to exhibit significant anti-inflammatory activities in the carrageenan-induced rat paw edema test and the fertile egg test[118].

**201**

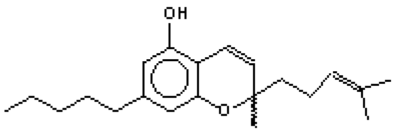

**Cannabichromene**

**202**

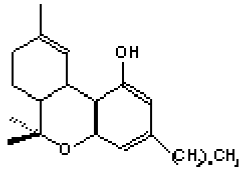

**Δ[1]-Tetrahydrocannabinol (Dronabinol)**

Cannabichromene has anti-inflammatory activity[77]. Δ[1]-Tetrahydrocannabinol, the active principle of marihuana, has anti-inflammatory, antiemetic, and hallucinogenic properties. Used topically in hypertensive glaucoma[119]. Isolated from *Cannabis sativa* (Cannabaceae) leaves.

**203**

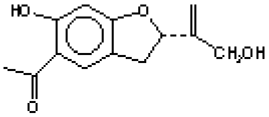

**Tremetone**

**204**

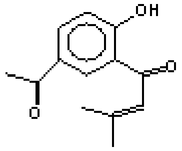

**Non-benzofuran acetophenone 1**

**205**

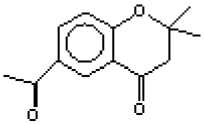

**Non-benzofuran acetophenone 2**

Isolated from *Ophyosporus axilliflorus* Griseb. (Asteraceae). Exhibited anti-inflammatory activity on carrageenan-induced mouse paw edema[120].

**206**

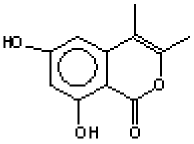

**6,8-Dihydroxy-3,4-dimethyl-**
**1H-2benzopyran-1-one**

**207**

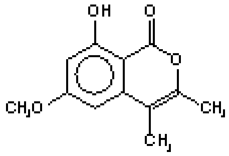

**Polygonolide**

Found in the roots of *Polygonum hydropiper* (Polygonaceae). Has anti-inflammatory activity[115,121].

**208**

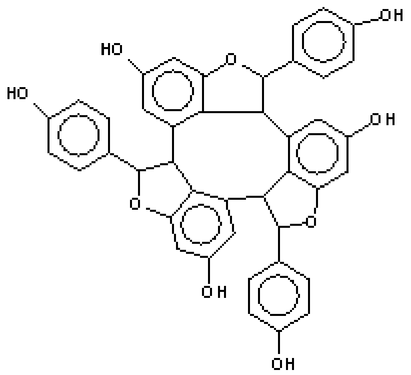

**Vineferin**

**209**

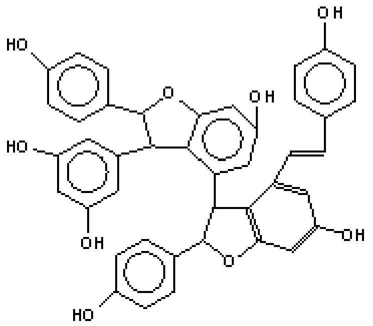

**Miyabenol**

**210**

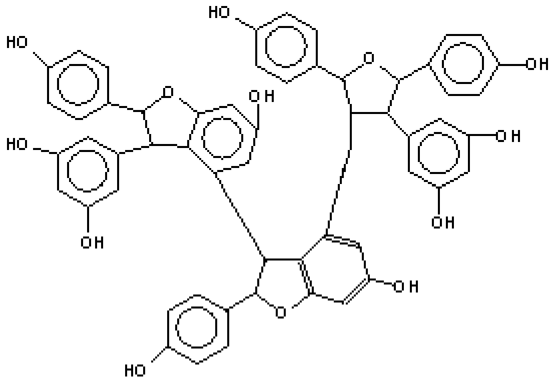

**Kobophenol**

These oligomeric stilbenes have been isolated from *Caragana sinica* (Buchoz) (Leguminosae). Exhibited protein kinase C inhibitory activity at low micromolar concentrations[122].

**211**

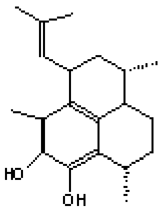

**8,10,12,14-Epiamphilectatetraene-9,10-diol**

A constituent of *Pseudopteregorgia elisabethae* and has anti-inflammatory activity[121].

## PHENYLPROPANOIDS

**212** **213** **214**

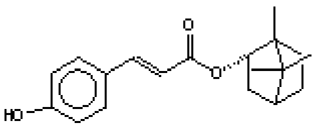

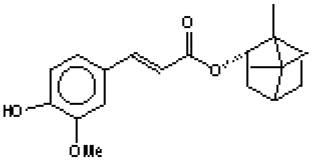

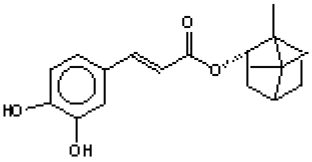

**(-)-Bornyl p-coumarate** **(-)-Bornyl ferulate** **(-)-Bornyl caffeate**

Isolated from the roots of *Coreopsis mutica* var. Mutica (Compositae). Have anti-inflammatory activity[123].

**215**

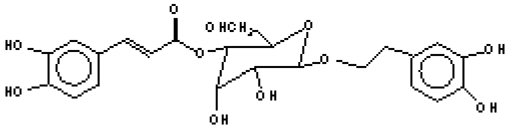

**Calceolarioside A**

Found in *Calcolaria hypericina* Poepp. (Scrophulaiaceae). Has anti-inflammatory activity[124].

**216**

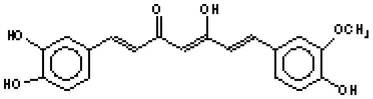

**Curcumin**

A constituent of turmeric. Has been shown to be an effective anti-inflammatory agent. As potent as phenylbutazone in the carrageenan edema test but half as potent in chronic tests. The mechanism of anti-inflammatory activity is found to be multifactorial. Inhibited lysosomal enzymes (acid phosphatase and cathepsin D) and was effective in inhibiting lipid peroxide formation[89]. Has cytotoxic and antioxidant activities. Reduces cholesterol level and helps control blood sugar. Isolated from rhizomes of *Curcuma longa*, *C. aromatica*, and *C. xanthorrhiza* (Zingiberaceae).

**217**

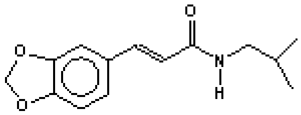

**Fagaramide (piperonyl-4-acrylicisobutylamide)**

Effective against carrageenan paw edema in rats and was approximately 20 times less potent than indomethacin. Effective against the prostaglandin phase of an acute experimental inflammatory reaction. It is thus suggested that at least part of the anti-inflammatory activity of fagaramide is mediated via inhibition of prostaglandin synthesis[125]. The roots of *Zanthoxylum zanthoxyloides* and *Fagara xanthoxyloides* (Rutaceae) have been used to treat allergic and anti-inflammatory diseases. The activity is due a fagaramide, a phenylpropanoid, that inhibits the edema of the rat paw induced by carrageenan[89].

**218**

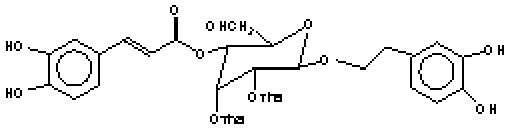

**Forsythoside**

Occurs in *Calcolaria ascendens* Lind. (Scrophulaiaceae). Has anti-inflammatory activity and enhanced production of cyclo-oxygenase products[124].

**219**

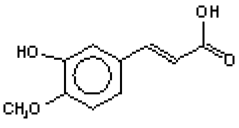

**Isoferulic acid**

Obtained from the crude extract of *Cimicifuga dahurica* Maxim. Ranunculaceae rhizomes reduce carrageenan-induced edema[106].

**220**

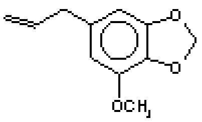

**Myristicin**

The fruit of *Myristica fragrans* Houtt (Myristacaceae) has been used in Indonesian folk medicine as aromatic stomachics, analgesics, and a medicine for rheumatism. The anti-inflammatory activity in carrageenan-induced edema in rats and vascular permeability in mice is due to the major principle myristicin[126].

**221**

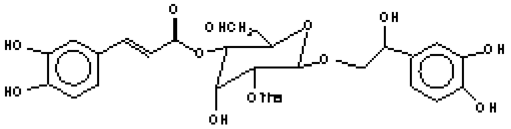

**Orobanchoside**

Occurs in *Orobanche hederae* Duby (Orobanchaceae). Inhibited the formation of 5-lipoxygenase product 5-HETE[124].

**222**

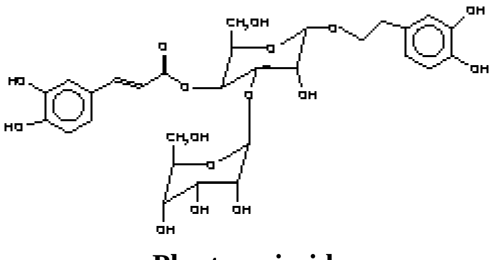

**Plantamajoside**

Occurs in the leaves of *Plantago major* and *P. asiatica* (Plantaginaceae). Inhibits 5-lipoxygenase and cyclic adenosine monophosphate diesterase, thus explaining the anti-inflammatory activity of *Plantago* plants[89].

**223**

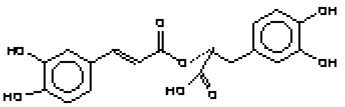

**Rosmarinic acid**

Occurs within the Labiatae in *Rosmarinus officinalis*, *Salvia officinale*, *Melissa officinalis*, *Menta piperita*, and *Teucrium scorodonia*. Also occurs in *Symphytum officinale* (Boraginaceae), in some Hydrophyllaceae and Acanthaceae, and in the genera *Anethum*, *Levisticum*, *Sanicula*, and *Astrantia* (Umbelliferae). Has anti-inflammatory activity[48].

**224**

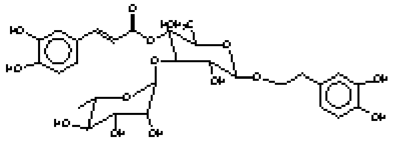

**Verbacoside**

The anti-inflammatory activity has been demonstrated on the exudative and proliferative phases of cotton pellet granuloma[48]. Isolated from leaves of *Buddleja globosa* and *A. officinalis* (Scrophulariaceae).

**225**

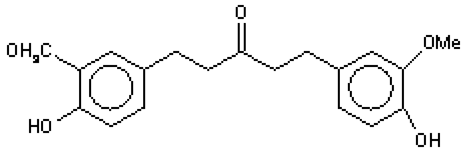

**1,5-Bis(4-hydroxy-3-methoxyphenyl)-(1E.4E)-1,4-pentadien-3-one**

**226**

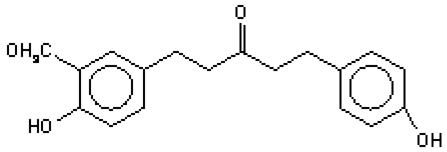

**1(4-Hydroxy-3-methoxyphenyl) 5-(4-hydroxyphenyl)-(1E.4E)-1,4-pentadien-3-one**

**227**

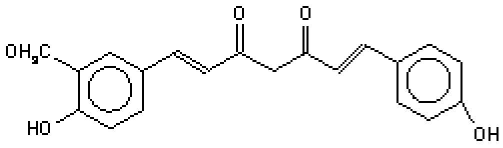

**Demethoxycurcumin**

**228**

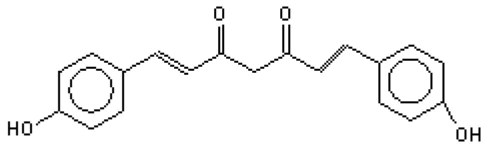

**Bisdemethoxycurcumin**

**229**

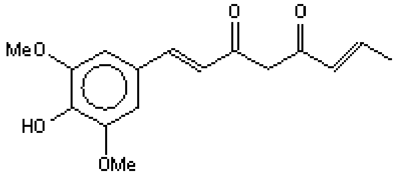

**5´-Methoxycurcumin**

Isolated from rhizomes of *Curcuma domestica* (Zingiberaceae). Showed anti-inflammatory activity[127].

## QUINONES

**230**

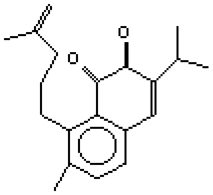

**Aethiopinone**

An o-naphthoquinone diterpenoid isolated from *Salvia aethiopis* L. (Labiatae) roots. Commonly used in folk medicine for a wide variety of remedies. Exhibited sizeable antinociceptive and anti-inflammatory effects in rodents. Produced a strong anti-inflammatory effect on an acute inflammatory process like that induced by carrageenan, which was in the same order of magnitude as that observed after poroxicam or ibuprofen administration and its antinociceptive effects were specially important against thermal painful stimuli[128].

**231**

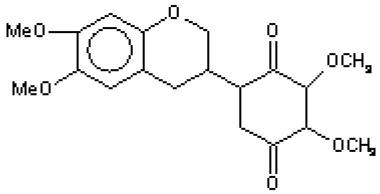

**Abruquinones A**

**232**

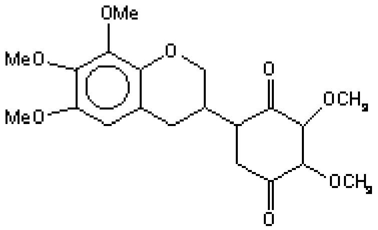

**Abruquinones B**

**233**

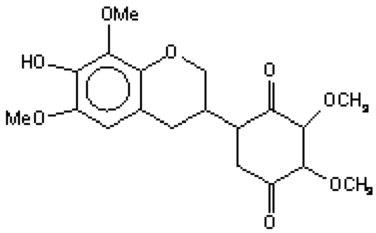

**Abruquinones D**

**234**

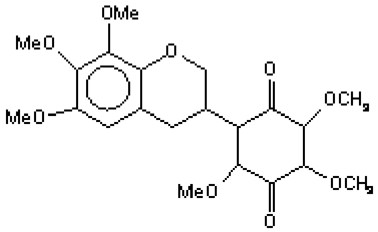

**Abruquinones E**

**235**

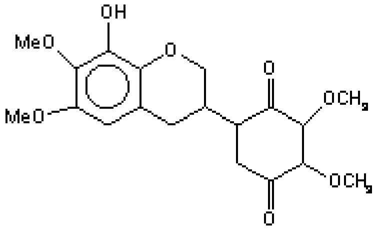

**Abruquinones F**

The pharmacological activities of the five isoflavanquinones isolated from the leaf of *Abrus precatorius* L. (Leguminosae) have been evaluated. The results indicate that abruquinones A, B, and D exhibited remarkable inhibitory effects on the platelet aggregation. The compounds A, B, D, and F showed strong anti-inflammatory and antiallergenic effects. The IC50 of abruquinones A, B, D, and F for the inhibition of superoxide formation were less than 0.3 μg/ml for the inhibition of the release of both β-glucuronidase and lysosyme from rat neutrophils[129]. This plant is used as a folk-medicine in China for the treatment of laryngitis, hepatitis, and bronchitis, indicating that it exhibits anti-inflammatory activity.

**236**

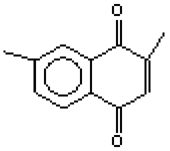

**Chimaphillin**

Anti-inflammatory and analgesic principles were isolated from the methanol extract of the whole herb of *Pyrola rotundifolia* L. (Pyrolaceae). Based on bioassays of the inhibitory activities on carrageenan-induced hindpaw edema in rats on acetic acid–induced writhing in mice. The principles were identified as ursolic acid and chimaphillin[88]. *P. rotundifolia* is found in the group of Chinese crude drugs called Qu-feng-shi-yao, which has long been used as a remedy mostly for arthritic diseases in traditional Chinese medicine.

**237**

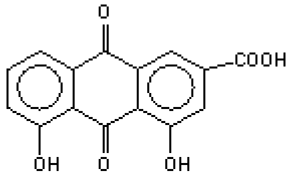

**4,5-Dihydroxyanthraquinone-2-carboxilic acid**

Possesses anti-inflammatory activity in carrageenan-induced paw edema. Isolated from *Rheum palmatum* leaves[121].

**238**

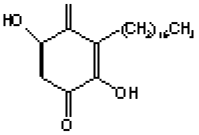

**Embellin**

Possesses anti-inflammatory activity in carrageenan-induced paw edema and cotton pellet granuloma formation[130]. Isolated from *Embelia ribes* Burm (Myrsinaceae) leaves.

**239**

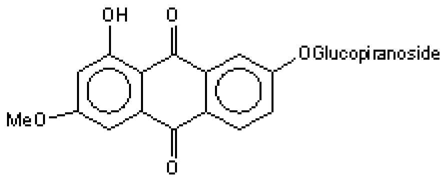

**8-Hydroxy-6-methoxy- 2 methylanthra-quinone-3-O-β-D-glucopyranoside**

Isolated from *Limonia crenulata* Roxb. (Rutaceae). Showed significant inhibition (65.9%) of carrageenan-induced paw edema in rats in a dose of 1 g/kg[131].

**240**

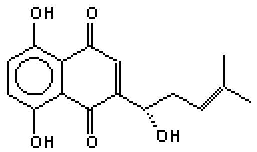

**Shikonin**

**241**

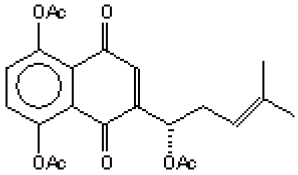

**Acetylshikonin**

Shikonin and acetylshikonin are active ingredients in the traditional Chinese drug Zi-Cao (*Arnebia euchroma* Royle, Boraginaceae). Both of these compounds inhibit the usual increase of capillary permeability induced in rats by histamine and have significant anti-inflammatory effects in rats on edema of the paws induced by acute exposure to formalin[132].

## TRITERPENOIDS AND STEROIDS

**242**

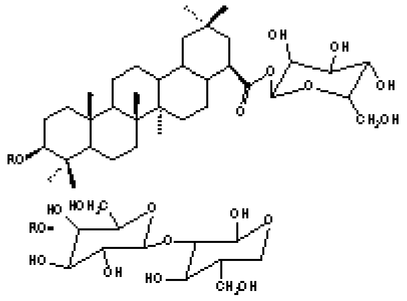

**Arvenoside A**

A triterpenoid saponin that showed strong anti-inflammatory activity[133,134]. Isolated from *Calendula arvensis* (Compositae) leaves.

**243**

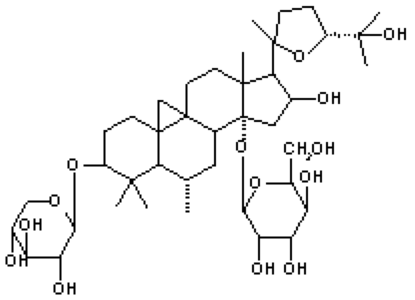

**Astramembrannin**

Isolated from Huang-Qi (the traditional Chinese drug plant *Astragalus membranaceus* Bge). Has anti-inflammatory action[135].

**244**

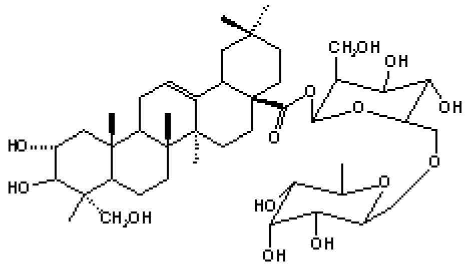

**Asteryunnanoside A**

**245**

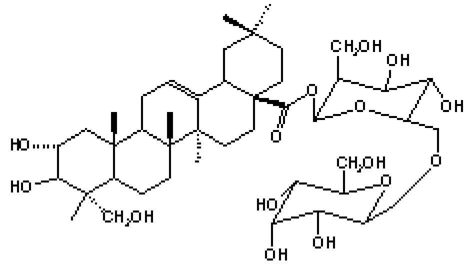

**Asteryunnanoside B**

**246**

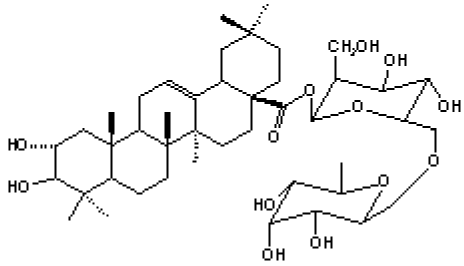

**Asteryunnanoside C**

Found in *Aster yunnanensis* (Compositae). Used as drug for the treatment of fevers, colds, tonsillitis, snake bites, and bee stings in Chinese medicine. Showed potent analgesic and anti-inflammatory activity[136].

**247**

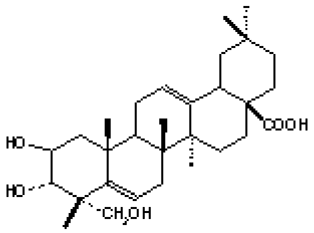

**Bassic acid**

Isolated from the ethanol extract of *Bumelia sartorum* Mart. (Sapotaceae) leaves and has been shown to be responsible for anti-inflammatory activity[133].

**248**

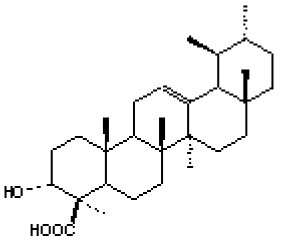

**Boswellic acid**

In India, the oleogum resin of *Boswellia serrata* Roxb. Ex Coleb (Burseraceae) has been released for therapeutic use under trade name “Sallaki” for the treatment of rheumatism and nervous diseases. It has been established that anti-inflammatory and antiarthritic activities are due to the presence of boswellic acid and other related pentacyclic triterpene acids[137].

**249**

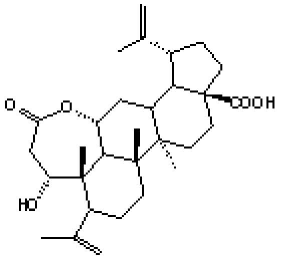

**Chiisanoside**

Used in Korean folk medicine, leaves and stem bark of *Acanthopanax chiisanensis* Nakai (Araliaceae) have been used as an antirheumatic. Chiisanoside, a glycosilated 3,4-secotriterpene of lupane-type with significant anti-inflammatory activity has been isolated from *A. chiisanensis*[138].

**250**

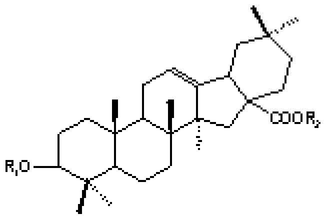

$R_1$= glucose (1→2)-glucoronic acid
$R_2$= glucose

**Chikusetsusaponin V**

The rhizomes of *Panax japonica* C.A. Meyer (Araliaceae) have been used for a long time in Japan as a substitute for *P. ginseng* roots of Chinese medicine. A saponin glucoside, chikusetsusaponin V isolated from the rhizomes of the plant, has shown significant anti-inflammatory activity[139].

**251**

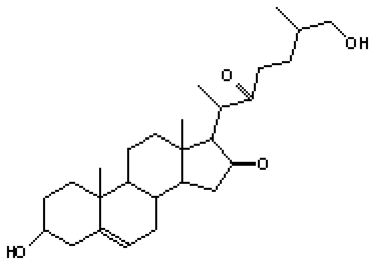

**Cryptogenin**

Obtained from *Dioscorea mexicana* (Dioscoreaceae) roots by extraction with ETOH. 80 mg/kg i.p. reduced kaolin edema in rats to half, 5 h after injection[140].

**252**

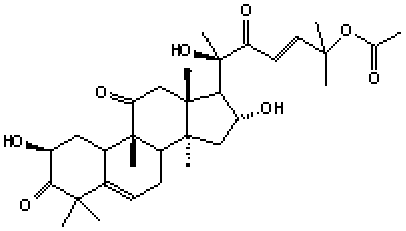

**Cucurbitacin**

The fruit juice of *Ecballium elaterium* L.A. Rich (Cucurbitaceae). Used in Turkish folk medicine for the treatment of sinusitis. Has been investigated for its anti-inflammatory activity. The active principle isolated from chloroform extract showed significant anti-inflammatory activity (69.6% inhibition of edema)[141].

**253**

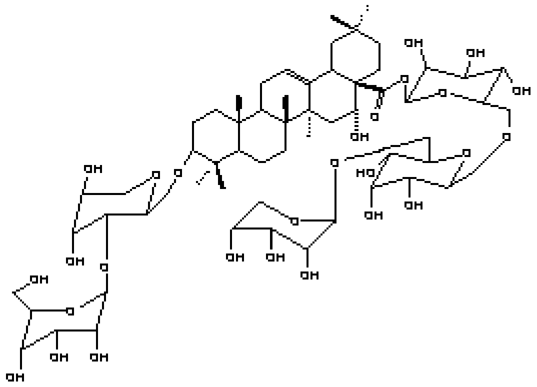

**Dulcin**

Found in *Pitchecellobium dulce* (Leguminosae). Showed anti-inflammatory activity[142].

**254**

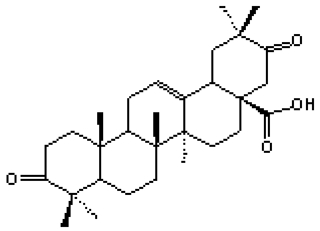

**Echinocystic acid**

Possesses activity capable of preventing various chemically induced edemas in rats and abdominal writhings following acetic acid injection in mice[143]. Isolated from *Baccharis trimera* (Asteraceae) leaves. This South American plant is used in traditional medicine, internally, for the treatment of rheumatism, hepatobiliary disorders, and diabetes.

**255**

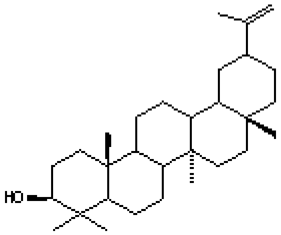

**3-Epibetulinic acid**

Showed anti-inflammatory activity[121]. Isolated from *Pricramnia pentandra* (Simarubaceae) leaves.

**256** **257** **258**

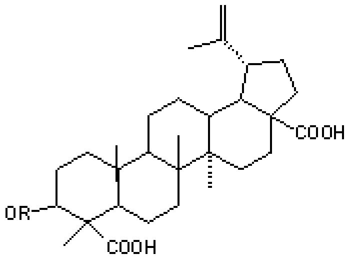

R=glc(6-1)glc(4-1)rha

**Fruticesaponin A**

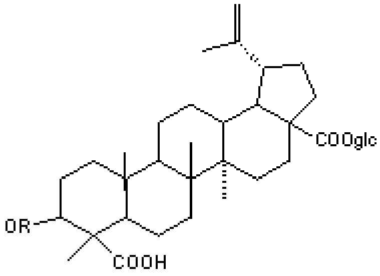

R= glc(4-1)rha

**Fruticesaponin B**

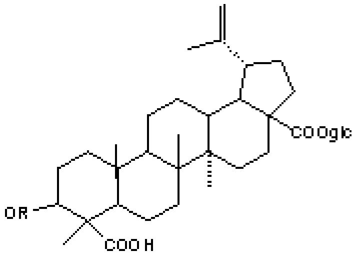

R=glc(6-1)glc(4-1)rha

**Fruticesaponin C**

Occur in the dry root of *Bupleurum fruticescens* (Umbelliferae). Listed officially in the Chinese and Japanese pharmacopoeias and used in Asian traditional medicine to treat certain disorders, are accompanied by inflammation. *In vivo* anti-inflammatory activity (antidematous activity)[144].

**259**

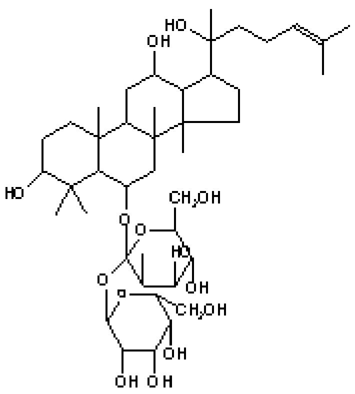

**Ginsenoside Ro**

An oleanane-type saponin that has been screened for activity in experimental models of inflammation. Inhibited an increase in vascular permeability in mice induced by acetic acid and reduced an acute paw edema in rats induced by carrageenan[145]. Isolated from roots of red *Panax ginseng* C.A. Meyer (Araliaceae). Widely prescribed in Chinese medicine in general practice and has been considered to be effective in the management of psychosomatic diseases such as indefinite complaints, stress ulcer, and anxiety neurosis. In addition, ancient literature indicates that ginseng root alleviates some types of inflammatory diseases.

**260**

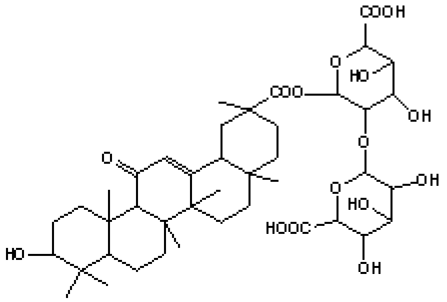

**Glycyrrhetinic acid glucoside**

**261**

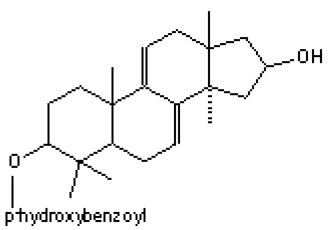

**3β-p-Hydroxybenzenzoyldehydrotumulosic acid**

Occur in the sclerotium of *Poria cocos* Wolf (Polyporaceae). Used in Chinese herbal prescriptions as a diuretic and a sedative. The 50% inhibitory doses of the compounds were 0.27 and 1.25 mg per ear on TPA- and AA-induced inflammation[146].

**262**

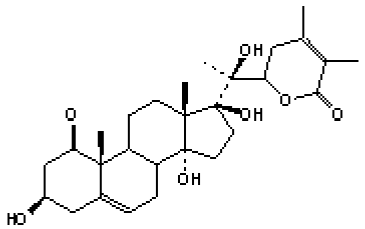

**3β-Hydroxy-2,3-dihydro-withanolide F**

Anti-inflammatory activity of 3β-hydroxy-2,3-dihydro-withanolide F has been assessed and has marked effects in subacute inflammation. A comparison of the anti-inflammatory properties showed it is approximately 5 times more active than phenylbutazone and equally active to that of hydrocortisone on weight basis. The withanolide did not exhibit any effect on CNS[147]. Isolated from *Withania coagulans* Dunal (Solanaceae) leaves. This plant is used in India for skin afflictions, inflammatory conditions, chronic liver complications, and as a sedative.

**263**

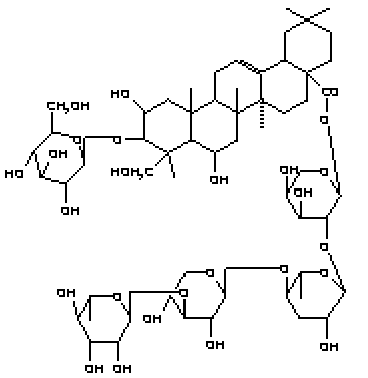

**Mi-saponin A**

A bisdesmoside of an olean-12 ene-type sapogenol protobassic acid. Obtained from seed kernels of *Madhuca longifolia* L. Macbride (Sapotaceae). The activity of anti-inflammatory activity was found to be ¼ of phenylbutazone[148].

**264**

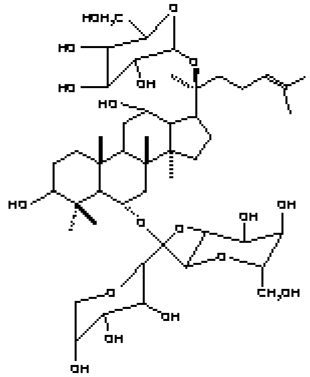

**Notoginsenoside R1**

Occurs in *Panax notoginseng* (Araliaceae). Has anti-inflammatory activity[149].

**265**

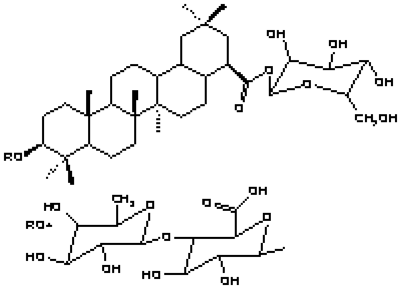

**Olaxoside**

Found in various *Olax* spp. (Olaneaceas). Showed strong anti-inflammatory activity[133,134].

**266**

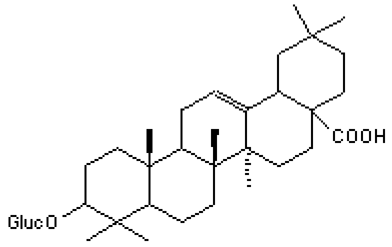

**Oleanolic acid 3-glucoside**

Showed significant antiarthritic activity in the exudative and proliferative phases of inflammation in rats[150]. Isolated from *Randia dumetorum* Lam. (Rubiaceae) seeds.

**267**

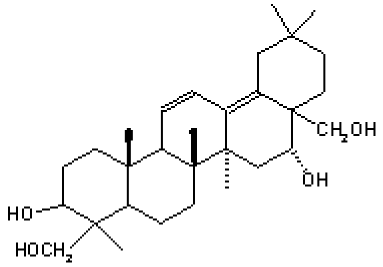

**11,13(18)-Oleanadiene 3,16,23,28, tetrol.**

A constituent of *Bupleurum falacatum*. An anti-inflammatory agent[8].

**268**

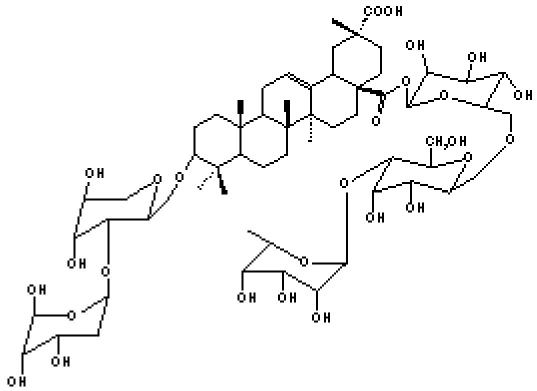

**3-O-[α-arabinopyranosyl-(1-2)-α-L-arabinopyranosyl]olean-12ene-28,29-dioic acid 28-[O-α-L-rhamnopyranosyl-(1-4)-D-glucopyranosyl-(1-6)β-glucopyranoside**

Found in the bark of *Nothopanax davidii* Harms (Araliaceae). Used in traditional Oriental medicine for more than 5000 years. Has anti-inflammatory activity[151].

**269**

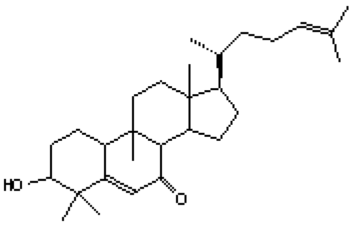

**7-0x0-10α-Cucurbita-5,24-dien-3β-ol**

Isolated from the seeds of *Trichosanthes kirilowii* Maxim. (Cucurbitaceae). Showed anti-inflammatory effect[152].

**270**

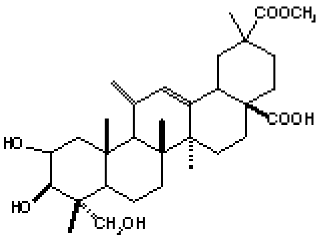

**Phytolaccoside B**

This saponin is a glucoside of jaligonic acid 30 methyl ether, exhibiting antirheumatic and anti-inflammatory actions[153,154]. Isolated from *Phytolacca americana* L. (Phytolaccaceae) roots.

**271**

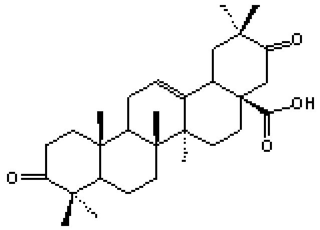

**Propapyriogenin $A_2$**

Obtained by acid hydrolysis of the saponin fractions of *Tetrapanax papyriferum* (Araliaceae) and *Bupleurum rotundifolium* (Umbelliferae). Showed strong anti-inflammatory activity[133,134].

**272**

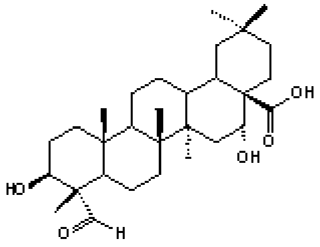

**Quillaic acid**

Obtained from the acid hydrolysis of the saponin fraction from the bark of *Quillaja saponaria* (Rosaceae). The saponin-rich bark of *Q. saponaria* (10%) is used in shampoo liquids. Shown to have analgesic and anti-inflammatory activity[141].

**273**

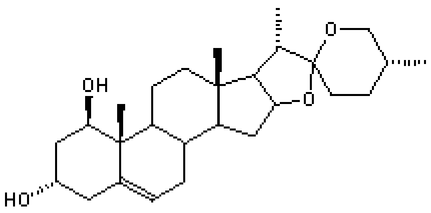

**Ruscogenin**

Occurs in *Ruscus aculeatus*. Used in the local treatment of hemorrhoids. Showed anti-inflammatory effect[8].

**274**

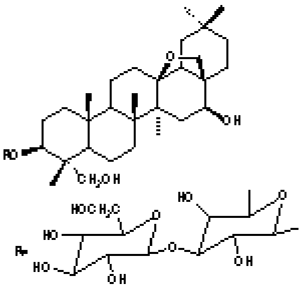

**Saikosaponin A**

The roots of *Bupleurum falcatum* L.B. chinense (Umbelliferae) are used in Chinese medicine for the treatment of inflammation of the diaphragm and enlargement of the liver caused by hepatitis. A number of saikosaponins have been isolated and the aglycone part was found to have oleanane-type structure with a characteristic unsaturation at C-11 and 13β,28-oxide system. The administration of saikosaponins has no side effect such as shrinking of the adrenal gland that often occurs with the administration of prednisolone[106].

**275**

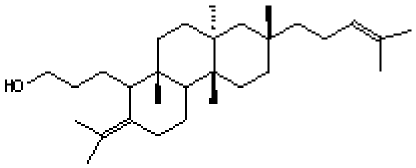

**Sasanquol**

Found in the seed of *Camellia sasanqua* Thumb (Theaceae). The 50% inhibitory dose of this compound against TPA-induced ear inflammation (1 μg per ear) in mice was 0.4 per ear[155].

**276**

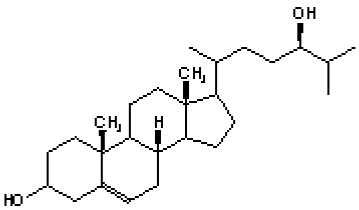

**β-Sitosterol**

The anti-inflammatory and antipyretic activities of β-sitosterol isolated from *Cyperus rotundus* (Ciperaceae) and *Bryophyllum pinnatum* (Crassulaceae) leaves have been studied, employing carrageenan-induced edema, cotton pellet implantation, and Brewer´s yeast-induced pyrexia in rats. β-Sitosterol was found to possess potent anti-inflammatory activity against both tests, similar to hydrocortisone and oxyphenbutazone when administered i.p.[156,157].

**277**

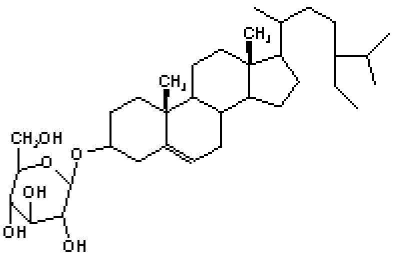

**β-Sitosterol-β-D-glucopyranoside**

**278**

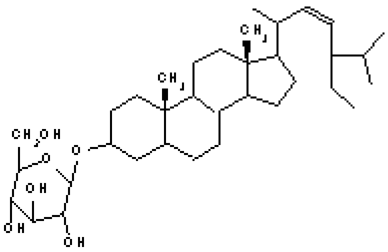

**Stigmasterol-β-D-glucopyranoside**

The isolated glucoside from *Sechium edule* (Cucurbitaceae) leaves was tested against phenylbutazone and the vehicle control using dextran-induced rat paw edema method[158].

**279**

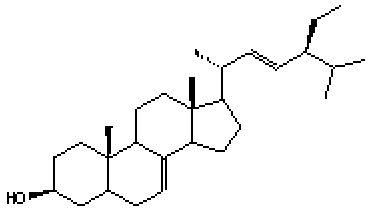

**α-Spinasterol**

Showed significant activity against acute inflammation induced by carrageenan in rats and was more potent than phenylbutazone but less potent than betamethasone[159]. Isolated from *Symplocos spicata* Roxb. (Symplocaceae) roots.

**280**

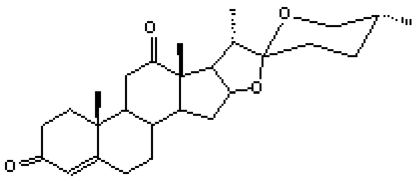

**25R-Spirostan-4-ene-3,12-dione**

Found in *Polygonum chinensis* L. Polygonaceae. Has anti-inflammatory and antiallergic activities[160,161]. Treatment of dysentery.

**281**

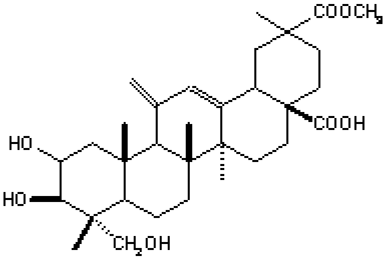

**282**

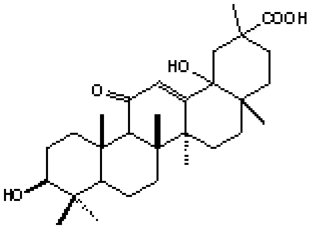

**283**

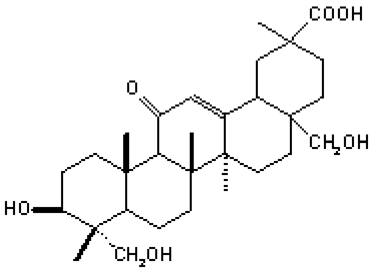

**Terminolic acid** **Glycyrrhezinic acid** **Lonchoterpene**

Terminolic acid, glycyrrhetinic acid, and lonchoterpene showed anti-inflammatory activity[162]. Isolated from *Terminalia ivorensis* (Combretaceae) bark. The glycyrrhezinic acid potentiates the antiarthritic action of hydrocortisone in rats and showed anti-inflammatory activity in 1/8 potency of cortisol by the cotton pellet method[133]. Isolated from *Glycyrrhiza glabra* Linn (Leguminosae) roots.

**284**

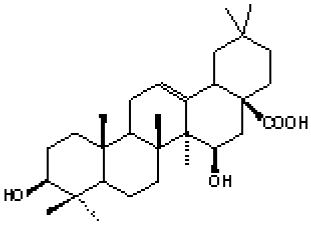

**Triptotriterpenic acid**

Showed anti-inflammatory activity[121]. Isolated from *Tripterygium wilfordil* leaves.

**285**

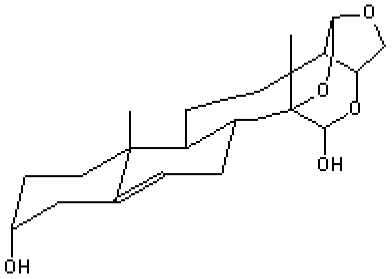

**Velutinol A**

Has antibradykinin, anti-inflammatory activity[163]. The structure of velutinol A comprises a novel pregnane skeleton, and steroids of this class are commonly encountered in Apocinaceae[164]. Extracted from *Mandevilla velutina* (Aponcinaceae) rhizomes.

**286**

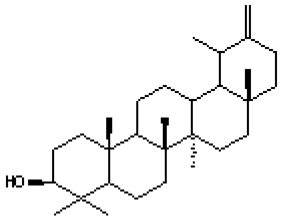

**Taraxasterol**

**287**

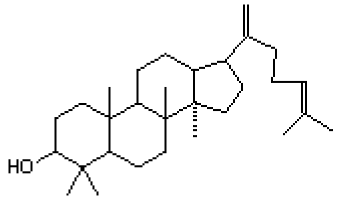

**Dammaradienol**

**288**

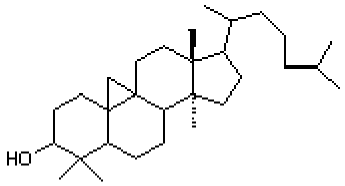

**Cycloartenol**

**289**

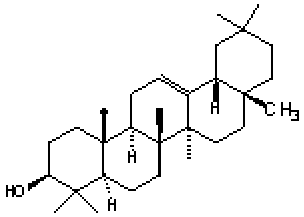

**β-Amyrin**

**290**

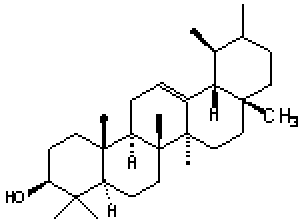

**α-Amyrin**

**291**

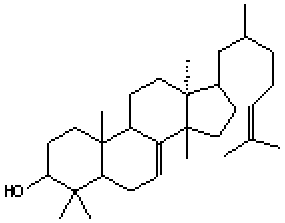

**Tirucalla-7,24-dienol**

**292**

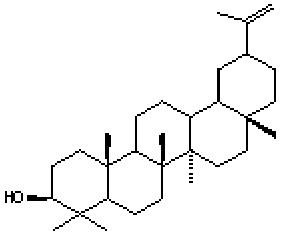

**Lupeol**

Six species of Compositae plants — *Calendula officinalis, Carthamus tintorius, Cosmos bipinnatus, Chrysanthemus morifolium, Helianthus annuus*, and *Matricaria matricarioides* — were investigated for their triterpene alcohol constituents. This led to the isolation and identification of 11 triterpene alcohols as follows: Helaniol, Taraxerol, Dammaradienol, β-amyrin, Cycloartenol, Tirucalla-7,24-dienol, α-amyrin, Lupeol, 24-methylenecycloartanol, ψ-taraxasterol, and Taraxasterol. Triterpene alcohols isolated from the Compositae flowers in this study were examined for their inhibitory effects on TPA-induced inflammation in mice. The inhibitory activities were compared with indomethacin and hydrocortisone. All of the triterpene alcohols markedly inhibited the TPA-induced inflammation[165]. Also inhibited eukaryote signal-regulated protein kinases[166].

**293**

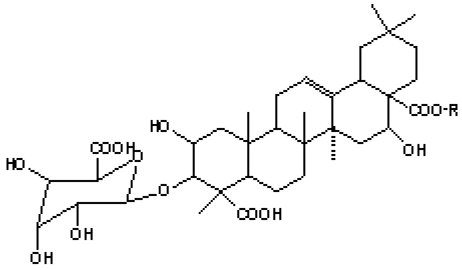

**Zanhasaponin A**

**294**

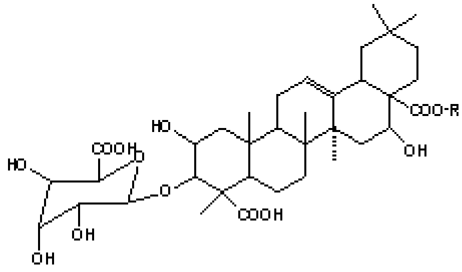

**Zanhasaponin B**

Found in *Zanha africana* root bark (Sapindaceae). Used in traditional medicine in Southern Africa for the treatment of dysentery. Have anti-inflammatory activity[161].

**295**

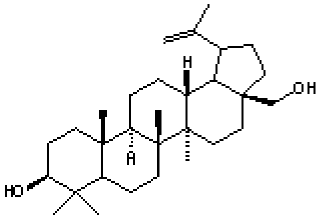

**Betulin**

**296**

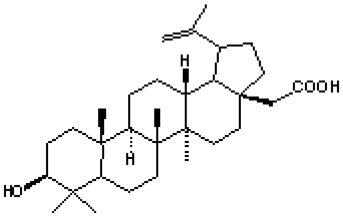

**Betulinic acid**

**297**

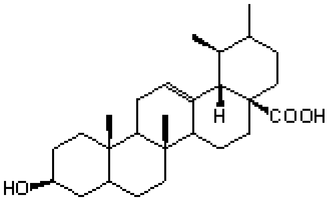

**Ursolic acid**

Three anti-inflammatory triterpenes — betulin, betulinic acid, and ursolic acid — were isolated from *Diospyros leucomelas* L. (Ebenaceae) leaves. They showed anti-inflammatory activity in the carrageenan and serotonin paw edema tests and TPA and EPP ear edema tests. The blockade of their effects by progesterone, actinomycin D, and cycloheximide has served to determine the mechanism of action in relationship with that of glucocorticoides[167].

## XANTHONES

**298**

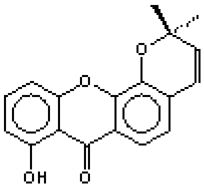

**Dehydrocycloguandin**

The anti-inflammatory activity has been demonstrated on the exudative and proliferative phases of cotton pellet granuloma, formaldehyde-induced arthritis, and carrageenan edema[168]. Isolated from *Calophyllum brasiliense* and *C. inophyllum* (Guttiferae) leaves.

**299**

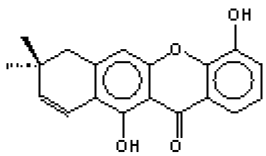

**6-Deoxyjacareubin**

Significantly inhibited carrageenan- and yeast-induced edema and cotton pellet inflammation[168]. Isolated from leaves of *Calophyllum zeylanicum* and *Kielmeyera speciosa* (Guttiferae).

**300**

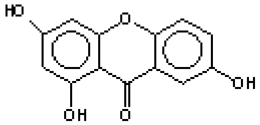

**Euxanthone**

A xanthone. Exhibited anti-inflammatory activity[169]. Isolated from seeds of *Mammea americana* (Guttiferae).

**301**

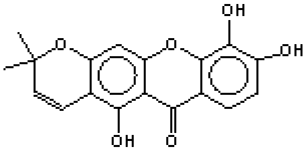

**Jacareubin**

Found in the wood of *Calophyllum brasiliense, Kielmeyera ferruginea*, and *Pentadesma butyracea* (all Gutiferae). Has anti-inflammatory activity[169].

**302**

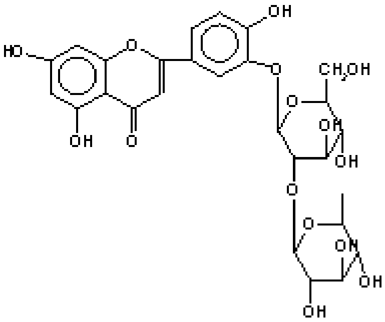

**Luteolin-4´-O-neohesperidoside**

Has significant anti-inflammatory action. More potent than ibuprofen. Isolated from *Caralluma attenuata* Wight (Asclepiadaceae)[55].

**303**

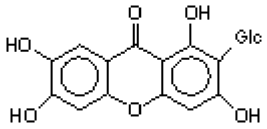

**Mangiferin**

**304**

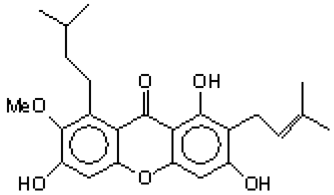

**Mangostin**

Xanthone mangiferin and phenylated xanthone mangostin have been found to exert pronounced anti-inflammatory activity when tested against different models of experimental inflammation[170]. Isolated from leaves of *Swertia chirata* Buch-Ham (Gentianaceae). This plant is used in the Indian system of medicine as an antimalarial, and liver tonic. Mangiferin exhibits significant anti-inflammatory properties at a dose of 50 mg/kg. They do not have analgesic or antipyretic effects but show antiulcer activity[169]. Isolated from leaves of *Canscora decussata* Schult (Gentianaceae) and *Cratoxylua prunifiorus* Kurz (Guttiferae).

**305**

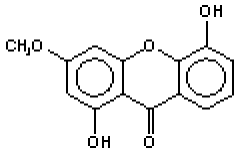

**Mesuaxanthone A**

**306**

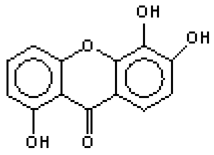

**Mesuaxanthone B**

The leaves of *Mesua ferrea* (Guttiferae) have been used to treat inflammatory diseases. The activity is due at mesuaxanthone A and B, that inhibited the increase in edema of the rat paw induced by carrageenan[168].

## ADDITIONAL REFERENCES

Claeson, P., Pongprayoon, U., Sematong, T., Tuchinda, P., Reutrakul, V., Soontornsaratune, P., and Taylor, W.C. (1996) Non-phenolic linear diarylheptanoids from *Cucurma xanthorrhiza*: a novel type of topical anti-inflammatory agents: structure-activity relationship. *Planta Med*. **62**, 236–240.

Pereira, B.M.R., Da Silva, B.P., Pereira, N.A., and Parente, J.P. (2000) Antiiflammatory and immunologically active polysaccharides of Periandra mediterranea. *Phytochemistry* **54**, 409–413.

Recio, M.C., Giner, R.M., Mañez, S., and Talens, A. (1995) Structural requirements for the anti-inflammatory activity of natural triterpenoids. *Planta Med*. **61**, 182–185.

Shibata, M., Yamatake, Y., and Amagaya, Y. (1975) Pharmacological studies on the Chinese crude drug "Shoma". Acute toxicity and antiinflammatory actions of *Cimicifuga rhizoma*, *Cimicifuga dahurica* Maxim. *Yakugaku Zasshi* **95**, 539–546.

### TABLE 1
### Mechanism of Action of Several Compounds Isolated from Plants with Anti-Inflammatory Activity

| | |
|---|---|
| A. Supress platelet function | Curcumin<br>Isoflavanquinones (abruinonones A, B, D, and F)<br>Diterpenoids (Aethiopinone)<br>Quinoid aporphine alkaloids<br>o-Naphthoquinone diterpene |
| B. Inhibit eicosanoid mediator generation or activities | The coumarins - herniarin, aesculetin, scopolin, and scopoletin |
| C. Block O2 radical formation or effects | Chamazulene<br>The coumaric, caffeic acid, ferulic acid, and derivatives (methyl caffeate, methyl feulate and bornyl ferulate)<br>(-) Epicatechin, (-) epiafzelechin, artemetin<br>Flavonoids (quercetin and kaempferol)<br>The flavan 3-ol derivatives, 4´-O-methyl-ent-gallocatechin, (+)-gallocatechin, mearnsitrin, myricitrin and quercitrin<br>6-Hydroxyflavonoids as 6-hydroxykaempferol, 3,6-dimethyl ether, and 6-hydroxyluteolin<br>The ocobullenone<br>Polyacetylenes<br>Sesquiterpene lactone hydroxyachillin |
| D. Alter cytokine production or effects | The pimarane diterpenes (2$\alpha$-acetoxysandara-copimaradien, maxikdiol and sandara-copimaradien-1$\alpha$,2$\alpha$–diol)<br>Sesquiterpene lactones (diversifolin, methyl ether, and tirotundin)<br>Sesquiterpene lactones of the germacranolide-type |

| | |
|---|---|
| | (melampolides and millerenolides) |
| E. Block lysosomal enzymes | Acid phosphatase and cathepsin D |
| | Flavonoids (hypolaetin-8-glucoside) |
| | Hypolaetin-8-glucoside |
| | Isoflavanquinones (abruinonones A, B, D, and F) |
| | Quercetin and kaempferol |
| F. Antagonize other low m.w. mediators | α-Amyrin and lupeol and the palmitate and linoleate esters of these triterpenoids |
| | Baicalein, baicalin, and wogonin |
| | Fagaramide, velutinol A |
| | Glycoside nepretin |
| | The kaempferol 3-O-sophoroside |
| | Lithocholic acid, 18α- and 18β-glycyrrhetinic acid, 5-cholenic acid-3β-ol, lithocholic acid methyl ester, ursolic acid, and oleanolic acid |
| | Nimbidin and bradykinin, benzoxazinoid |
| | The oligomeric stilbenes [(+)-α-viniferin, miyanenol, and kobophenol] |
| | The saponin echinocystic acid |
| | Sesquiterpene lactone (limonin) |
| | Sesquiterpene lactones (diversifolin, diversifolin methyl ether, and tirotundin) |
| | Several phenolic diarylheptanoids |
| G. Inhibit leukocyte function | Bukittinggine |
| | Epicatechin, catechin procyanidin $B_2$, |
| | 5,7 Dimethoxyflavone |
| | Gossypetin-8-glucoronide (hibifolin) |
| | The oleanane-type triterpene saponins zanha-saponins A and B |
| | Hypolaetin-8-glucoside |
| | Several triterpenoids belonging to the lupane, Oleanane and ursane series |
| | Procyanidin $B_4$, cinnamonol $D_1$ and cinnamonol $D_{2,}$ |
| | Quercetin glycosides |
| H. Other effects | Acetophenones |
| | Anthocyanins |
| | Benzophenanthridine (BPA) alkaloids (sanguinarine, chelerythrine, and chelidonine) |
| | Clerodane diterpene:19-nor clerodane diterpene |
| | The bicycle form of sesquiterpene molecule with four and nine members rings (β-caryophyllene) |
| | Non-phenolic diarylheptanoids |
| | The pimarane diterpenes (2α-acetoxysandara-copimaradien, maxikdiol and sandara-copimaradien-1α,2α–diol) |
| | The betulinic acid and betulin |
| | The ingenase-type diterpene structurally related to phorbol |
| | Lupeol |
| | The triterpenes - saponin, glycyrrhizin, and glycyrrhetic acid |
| | α-Spinasterol |
| | Saponin echinocystic |
| | Vitexin |

**TABLE 2**
**Applications of Several Compounds Isolated from Plants with Anti-Inflammatory Activity**

| Compound | Administration | Uses |
|---|---|---|
| Acetophenones | p.o. | Used in folk medicine in Argentina for its anti-inflammatory properties. |
| Aescin (β-amyrin derivative) | p.o. | Used as a folk medicine in France and the tincture of this drug has been used for haemorrhoid, and in the treatment of varicose vein. |
| Aesculetin | p.o. | Used in Spain as a folk remedy against a variety of diseases such as inflammatory complaints and to achieve beneficial effects on the digestive tract. |
| α-Amyrin | p.o. | Used as a remedy for arthritis. |
| 3-O-[α-L-arabinopyranosyl-(1-2) -α-L-arabinopyranosyl]olean-12-ene-28,29dioic acid[O-α-L-rhamnopyranosyl-(1-4)β-D-glucopyranoside. | p.o. | Show various pharmacological activities including increasing mental efficiency, recovering physical balance, stimulation of metabolic function, and other general health promoting effects. |
| Anemonin and protoanemonin | p.o. | Have commonly been used as analgesic, diuretic, and anti-inflammatory agents. |
| Arachidonic acid | p.o.<br>topically | Used in South America in traditional medicine internally for the treatent of rheumatism, hepatobiliary disorders, and diabetes; externally, in cases of skin ulceration and wounds. |
| Artemetin | topically | Used in Brazilian folk remedies as anti-inflammatory and cicatrizing agents. |
| Baicalein, baicalin, and wogonin | p.o. | Used for treatment of some types of dermatitis, diarrhea, and various inflammatory diseases as an antiphologistic and antipyretic in traditional Chinese medicine. |
| Bassic acid | p.o. | Used in Brazilian folklore in the treatment of inflammatory disorders. |
| Benzoxazinoid | p.o. | Used in China for a long time for the treatment of rheumatism and neuralgia. |
| Bergenin | topically | An ingredient of gagles, tooth powders, and lotions used for eye troubles, muscular pains, and sores. |
| Boswellic acid | topically | Used in Ayurvedic system of medicine “Salai guggal” in India. Effective in controlled clinical trials in arthritis patients. Released for therapeutic use under the trade name “Sallaki”. |
| Brazilin and hematoxylin | p.o. | Have long been used as emmenagogue, as analgesic, as astringent antidiarrhoic, and as cure for contution and sprain in Oriental medicine. |
| Bukittinggine | p.o. | Used in West Sumatra, Indonesia to treat bronchial asthma and to relieve pain. |
| Calophyllolide | i.p. and p.o. | Used for the treatment of rheumatism, sore, eyes, scabies, gout in the Indian system of medicine. |
| Chamazulene | p.o. | The preparations from chamomile are used in the treatment of inflammatory diseases of skin and intestinum. |
| Chikusetsusaponin V | p.o. | Long used in Japan as antitussive and expectorant. |
| Chimaphilin | p.o. | Found in the group of Chinese crude drugs called Qu-feng-shi-yao, which has long been used as a remedy mostly for arthritic diseases in traditional Chinese medicine. |
| Cis-communic acid | topically | Traditionally used in Chinese medicine for the treatment of eczema, eruption, swelling, injury, etc. by topical application. |
| Cryogenin and nesodine | p.o. | Shown to exert significant anti-inflammatory activity in different disorders. |
| Cucurbitacin B | p.o. | Used as a folk medicine in Turkey for the treatment of sinusitis and arthritis. |

| | | |
|---|---|---|
| Curcumin | p.o. and topically | Should be a useful antiarthritic drug without the side effects commonly encountered with other nonsteroidal anti-inflammatory drugs. Used as agent for local application on infected wounds including malignancies. |
| Cyanidanol | p.o. | Used in Europe for the treatment of liver diseases (for 20 years). |
| Dauricine | p.o. | Used in traditional Chinese medicine for testing rheumatism. |
| Trans-dehydrocrotonin | p.o.<br>topically | Widely known in traditional phytotherapy for the treatment of diabetes, diarrhea, gastrointestinal disorders, and liver diseases. Used in the Amazon region (Brazil) in the form of tea or powdered and dried pills. |
| 1,7-Diarylheptanoids | topically | In Thailand, commonly used externally for the treatment of acne and skin inflammations. |
| 5,7 Dimethoxyflavone | p.o. | Used in the folk medicine of Thailand as a remedy for arthritis. |
| Ephedrine | p.o. | The crude drug mao is famous for containing alkaloids of the ephedrine series having sympathomimetic, antiallergic activity, perspiratory, and antitussive. |
| Flavonoid glycoside | p.o. | Used for the treatment of injury. |
| Flavonoids | p.o.<br>topically | A traditional medicine in China (jiangxiang). Used to treat blood disorder, ischemia, swelling, necrosis, and rheumatic pain. |
| Ginsenoside Ro[1] | p.o. | Widely prescribed in Chinese medicine in general practice. Effective in the management of psychosomatic diseases such as indefinite complaints, stress ulcer, and anxiety neurosis. |
| Germacranolides | p.o. | Used in the Philippines for sore eyes, coughs, infections of stomach and intestines, itching and for snake and scorpion bites. |
| Glycyrrhetinic acid | p.o. | Used in African ethnomedicine for treatment of various diseases including arthritic conditions. |
| Glycyrrhizin | p.o. | Used in Western and Oriental medicine as an expectorant, antitussive, and as a sweetening adjuvant. |
| Gossypin | p.o. | Used as a remedy for arthritis. |
| Helenalin | topically | Used as a remedy for arthritis. |
| 3β-Hydroxy-2,3-dihydro-withanolide F | p.o. | Used in India for a number of ailments especially in skin infections, inflammatory conditions, asthma, chronic liver complications, and as sedatives. |
| 3β-Hydroxybenzoyldehydrotu-mulosic acid | p.o. | Used in Chinese prescriptions as a diuretic and as a sedative. |
| Hydroxyachillin | p.o. | Used since ancient times in Spanish traditional medicine as an anti-inflammatory and antirheumatic agent, and for their benificial effects on the digestive tract. |
| Hypolaetin-8-O-glucoside | p.o. | Employed in Spanish folk medicine as an anti-inflammatory and antirheumatic agent. |
| Isoflavanquinones | p.o. | Used in China for the treatment of laryngitis, hepatitis, and bronchitis. |
| Kaempferol | p.o. | Used as antipyretic, refrigerant, useful against thirst, leprosy, and leucoderma. |
| Lignans and neolignans | p.o. | Long used for treatment of headaches, as a tranquilizer and analgesic, and is considered to be especially efficacious for empyem, chronic inflammation of paranasal sinuses. A traditional Chinese herbal medicine know as “Shin-i”. |
| Limonin | p.o. | Used for treatment of headache, thoracicoabdominal pain, and vomiting, as an analgesic agent or cold remedy, and as an improving agent of blood circulation in traditional Chinese medicine. |
| Lupane saponins | p.o. | Listed officially in Chinese and Japanese pharmacopoeias and used in Asian traditional medicine to treat certain disorders thar are accompanied by inflammation. |
| Magniferin | p.o. | Used as a remedy for arthritis, and antimalarial and liver tonic. |

| | | |
|---|---|---|
| Marmin | p.o. | Used as antidiarrhoeitic and antidote to snake venom. |
| Maslinic acid | topically | Topical application traditionally used for the treatment of prickly heat, eczema, and other skin diseases. |
| Nimbidin | p.o. | Possesses antigastric ulcer activity, prevents ulceration by reducing gastric secretion, useful as a liniment for rheumatic affections. |
| Ocubellenone | p.o. | Used in the traditional medicine of Southern Africa for headaches, injury, and magical purposes. |
| Osthol | p.o. | Found in the Chinese crude drug called "Qu-feng-shi-yao"; used as a remedy for arthritis diseases, lumbago, edema, and common cold. |
| Phenanthrene derivatives | topically | Used in the folk medicine in Paraguay as anti-inflammatory when applied topically. |
| Phytolaccoside B | p.o. | Used in Korean medicine to alleviate rheumatism. |
| Polyacetylenes | p.o. | A traditional remedy in Hawaiian folk medicine for treating general debility of the body, throat, and stomach disorders, and of asthma. |
| Polysaccharides | p.o. | Widely used in Brazilian ethnomedicine as a cough suppressant, anti-inflammatory, and expectorant. |
| Quillaic acid | p.o. | Used as a analgesic and as a anti-inflammatory. |
| Saikosaponins | p.o. | Used in Chinese medicine for the treatment of inflammation of diaphragm and enlargement of liver caused by hepatitis. |
| Sesquiterpenes | p.o. | Used as a remedy for arthritis diseases. |
| Sesquiterpene Lactones | p.o. | Used in Central America as a remedy for skin infections. |
| Sinoacutine | p.o. | Used in the folk medicine in South Africa; this alkaloid exhibited slight anti-inflammatory activity; used as a remedy for arthritis. |
| β-Sitosterol | i.p. | Used for the treatment of rehumatism. |
| Taspine | p.o. | Used for the treatment of rheumatism by the Peruvian Indian. |
| Trilobine and isotrilobine | p.o. | Used for the treatment of rehumatism. |
| 3,4-Seco triterpene | p.o. | Used as an antirheumatic, anti-inflammatory, and tonic in Korea. |
| Ursolic acid | p.o. | Used in traditional Chinese medicine only for arthritic diseases. |
| Velutilol A | i.p. | Used as a folk medicine in Brazil; bradykinin antagonist; possesses low oral activity and very short-lived systemic activity; used as an analgesic, anti-inflammatory, and as an antirheumatic. |
| Vitexin | p.o. | Exhibited moderate anti-inflammatory activity; used as anti-inflammatory, hypotensive, and antispasmodic . |
| Zanhasaponins A and B | p.o. | Used in traditional medicine in Southern Africa for the treatment of dysentery. |
